# The Interaction of Factor Xa and IXa with Non-Activated Antithrombin in Michaelis Complex: Insights from Enhanced-Sampling Molecular Dynamics Simulations

**DOI:** 10.3390/biom13050795

**Published:** 2023-05-06

**Authors:** Gábor Balogh, Zsuzsanna Bereczky

**Affiliations:** Division of Clinical Laboratory Science, Department of Laboratory Medicine, Faculty of Medicine, University of Debrecen, 4032 Debrecen, Hungary; balogh.gabor@med.unideb.hu

**Keywords:** antithrombin, coagulation factor X, coagulation factor IX, protein–protein interaction, protein docking, molecular dynamics, conformational change

## Abstract

The interaction between coagulation factors Xa and IXa and the activated state of their inhibitor, antithrombin (AT),have been investigated using X-ray diffraction studies. However, only mutagenesis data are available for non-activated AT. Our aim was to propose a model based on docking and advanced-sampling molecular dynamics simulations that can reveal the conformational behavior of the systems when AT is not binding a pentasaccharide. We built the initial structure for non-activated AT-FXa and AT-FIXa complexes using HADDOCK 2.4. The conformational behavior was studied using Gaussian accelerated molecular dynamics simulations. In addition to the docked complexes, two systems based on the X-ray structures were also simulated, with and without the ligand. The simulations revealed large variability in conformation for both factors. In the docking-based complex of AT-FIXa, conformations with stable Arg150–AT interactions can exist for longer time periods but the system also has a higher tendency for reaching states with very limited interaction with the “exosite” of AT. By comparing simulations with or without the pentasaccharide, we were able to gain insights into the effects of conformational activation on the Michaelis complexes. RMSF analysis and correlation calculations for the alpha-carbon atoms revealed important details of the allosteric mechanisms. Our simulations provide atomistic models for better understanding the conformational activation mechanism of AT against its target factors.

## 1. Introduction

Antithrombin (AT) is a protease inhibitor glycoprotein in the serpin superfamily and one of the main regulators of the coagulation cascade [1,2,3]. It can inactivate its main targets, factor Xa (FXa), factor IXa (FIXa) and thrombin, through an irreversible serpin mechanism. In the presence of heparin analogs, the rates of inactivation are enhanced by at least three orders of magnitude [4].

The binding of a high-affinity pentasaccharide sequence in heparin chains triggers complex series of conformational changes in AT [4,5,6]. The first step mainly affects the heparin binding site, while the second step has consequences on several parts of the molecule. It has been demonstrated that the expulsion “hinge” region, the N-terminal end of the reactive center loop (RCL), takes place only in the third step from its closed position in β-sheet “A” [5,7]. Another important mechanism is the formation of a “bridge” between AT and the coagulation factor through a longer heparin chain, this playing a critical role especially in the inactivation of thrombin [4,8].

The AT–FXa and the AT–FIXa interaction has been extensively studied previously using X-ray diffraction [9,10], mutagenesis [11,12] and enzyme kinetics [5,6]. The X-ray diffraction experiments revealed the details of interaction between AT and the two coagulation factors in the Michaelis complexes at an atomic level. The two AT-inactivated factors’ complex structures are similar to each other in several important ways [9,10]. First, both structures show AT in a conformation fully activated by a pentasaccharide ligand, with the amino acids forming the “hinge” region located at a significant distance from the “A” β-sheet. Second, the position of the factors relative to AT is similar in the two structures. Third, the autolysis loop of the two factors, and especially the Arg150 amino acid according to the chymotrypsin numbering, forms interactions with a second binding site of AT outside the RCL, the “exosite”. However, the “static” X-ray diffraction structures provide very little information about the dynamic nature of protein–protein interactions. Furthermore, no experimental structure is available for the FXa and FIXa complexes of non-activated AT, and the available crystallographic data may not accurately describe the interactions in this state.

Kinetic experiments with engineered AT or factor variants also contributed significantly to the understanding of the mechanisms of these interactions. These studies have not only revealed the amino acids involved in the interaction, but also the dependence of their contribution on the presence of heparinoids, and thus the activation state of AT [7,11]. Izaguirre et al. investigated the roles of five AT residues of the antithrombin “exosite” by mutating one or more of them to alanine [12]. Using kinetic measurements, they classified these amino acids into two groups. Tyr253 and His359 play a critical role in the interaction regardless of the activation state while Asn233, Arg235 and Glu237 have a positive contribution in the activated states, but form repulsive interactions when no pentasaccharide or heparin chain is present (the numbering for AT residues corresponds to their position in the mature protein). They have also suggested that the expulsion of the “hinge” region may not be the single critical factor in the enhancement of the inactivation rates by the binding of heparinoids. Further studies have shown that AT also has an “intermediate activated” state with significantly increased activity against FXa and FIXa, but with a closed “hinge” region conformation [13,14]. Bonde et al. have studied the role of the autolysis loop of factor X, a region with a critical role in the AT interaction as well as other functions of the factor, by introducing artificial glycosylation sites using mutagenesis. The authors found that adding such glycans to the loop affects many aspects of factor Xa’s biochemistry, including activation, procoagulant activity and inhibition by antithrombin [15].

Molecular modeling, especially molecular dynamics simulations, can provide further insights into the behavior of protein–protein complexes in addition to the available experimental data. Such simulations can reveal biochemically interesting states with no atomistic experimental structure available. Furthermore, these techniques provide a means to investigate the dynamics of these systems and can predict multiple different conformations. However, long MD simulations for relatively large protein–protein complexes have very significant computational costs. As an alternative, enhanced-sampling molecular dynamics techniques have been developed. Using such techniques, the exploration of multiple conformational states of such systems might be achieved in relatively modest simulation times. Techniques that do not require pre-defined reaction coordinates, such as Gaussian accelerated molecular dynamics (GaMD) [16,17], are particularly well-suited for such goals.

Several molecular dynamics-based studies have been published for AT, investigating multiple aspects of the structure–function relationships, including the role of Asn135 glycosylation [18], the conformational behavior of the RCL in solution, the binding of the ligand with a different scaffold [19], the early events of ligand binding at an atomistic resolution [20] and the consequences of missense mutations [21,22]. Factor Xa and IXa were also the subject of significant research using MD-based methods. For the former, the binding of longer GAG chains has also been investigated, providing insights into the role of longer heparin chains of AT–FXa interactions [23]. However, to the best of our knowledge, this is the very first study of AT–FXa an AT–FIXa Michaelis complexes involving MD simulations.

The aim of this work was to build model systems using protein–protein docking and the enhanced-sampling MD method, GaMD simulations that can reveal the relevant binding conformations of FXa and FIXa to AT in non-activated conformations of the latter. We have built systems containing the closed “hinge” region conformations of AT, and investigated the conformational stability of these states using GaMD. We have also compared the results with those obtained through simulations based on the X-ray diffraction structures of the activated AT-factor complexes. We have also simulated these structures with the pentasaccharide ligands removed. Such simulations have provided insights into the conformational mechanisms of AT at an atomistic resolution. Our simulations provide a model for AT–FXa and AT–FIXa interactions in the Michaelis complexes where AT is in an activation state not observed in the available X-ray diffraction structures of the complexes.

## 2. Materials and Methods

In this paper, we apply chymotrypsin numbering for the factors, and the numbering of AT starts at the N-terminus of the mature protein. We built three types of model systems, both for factor Xa and IXa. Two models (for both factors) were based on the complex X-ray diffraction structures (2GD4 [9] for FXa, and 3KCG [10] for FIXa), with the pentasaccharide in silico removed from one of the two for investigating the role of the ligand in the interaction. The third system for both FXa and FIXa was built using structures obtained from protein docking. These models, unlike the X-ray structures, contained a “closed” conformation of the “hinge” region of the AT. The docking-based systems contained no pentasaccharide ligand.

Docking was performed using the HADDOCK 2.4 software, available as a cloud-based service [24,25,26]. The structures for the coagulation factors were derived from the two X-ray structures mentioned previously. Due to the low performance of the molecular dynamics simulations, we needed to reduce the sizes of the simulated systems. Therefore, the missing domains (GLA and EGF1) and amino acids in the light chains of the factors were not taken into account in the docked structures.

We selected a suitable initial conformation for the AT RCL from a previously published Gaussian accelerated molecular dynamics (GaMD) simulation from our group, based on system 1NQ9 [22]. The “optimal” structure was chosen based on the RMSD of Cα atoms in AT-reactive center loop (RCL) amino acids (residues 386–398) instead of the X-ray structure. The reason for this was that no X-ray structure of non-complexed AT has a suitable conformation of this loop, due to in-crystal contacts or engineered disulfide bonds [27,28]. In the HADDOCK docking, we applied two types of interaction restraints: “unambiguous” and “ambiguous”. Using “unambiguous” restraints, we fixed the following distances: the cleaved peptide bond in AT (Arg393–Ser394) and the catalytic center of the enzyme, the Arg393 side chain in AT and its binding pocket in the factor, as well as the distance of the Arg150 amino acid of the factor from the “exosite” of AT. The amino acids involved in “ambiguous” restraints were defined using the distance from the binding sites (AT: 233–235, 237, 251–253, 319, and 391–395;FXa: 40–42, 57, 99, 142–145, 148–152, 189–196, 213–221, and 225–228;FIXa: 41–42, 57–58, 99, 141–145, 148–152, 189–195, 213, 215–221, and 225–228). The HADDOCK software automatically performs clustering on the generated structures. The structure with the highest score according to the scoring algorithm of HADDOCK [29] from the best-ranked cluster was selected for the molecular dynamics simulation.

The solvated protein complex systems were built with the help of the CHARMM-GUI webserver [30,31]. The CHARMM36m force field was used for the proteins in the model systems [32]. Like in our previous works, we chose idraparinux as the ligand in models containing a pentasaccharide, modeled using the CHARMM carbohydrate force field [20,33]. The CHARMM version of the TIP3P water model [34] was used as a solvent model. All systems contained Na^+^ and Cl^−^ ions, with an approximate ionic strength of 0.15 M.

The AMBER 20 pmemd.cuda software was used for all molecular dynamics simulations [35,36]. The energy of solvated systems was minimized in multiple steps. First, 2000 steps of steepest-descent minimization were run with the non-solvent atoms restrained. This was followed by running 500 steps of the steepest descent and 1500 steps of the conjugate gradients algorithm without restraints. The systems were then heated to 310 K in a 2 ns NVT simulation and were pressure equilibrated for another 2 ns.

For system preparation, we performed conventional molecular dynamics (cMD) on all systems before the production simulations. For the systems based on the docked structures, the total length of cMD was 250 ns, with the “exosite distance” (between Arg150 in the two factors and Tyr253 in AT) fixed in the first 150 ns part. For all other models, a single 150 ns simulation was run without restraints.

The GaMD method was used for production simulations [16,17]. This technique can enhance conformational sampling without requiring any pre-defined reaction coordinates, which can be advantageous for protein–protein systems with a large conformational space. The equilibration phase of the GaMD simulations was 100 ns long. In the first 20 ns part, no acceleration was applied, with data collection occurring between 10–20 ns. In the remaining 80 ns, a regularly updated GaMD potential was added by the MD engine. From each GaMD equilibration run, two independent production simulations were started for each type of system, amounting to 4 simulations in total. We performed four independent 400 ns simulations for the systems based on docking, and two 300 ns and two 200 ns runs for the rest of the systems. The performed simulations are summarized in Table S1.

The systems were simulated under NVT conditions with a 2 fs integration timestep. The Langevin thermostat was used for temperature coupling, with a 1 ps^−1^ time constant. The Coulomb and Lennard-Jones (L-J) cutoffs were set to 12 Å. An L-J force switch was applied between 10 Å and the cutoff, as required for CHARMM force fields. Long-range electrostatic interactions were calculated using the PME method [37]. The default value of 6.0 was used for the sigma0P and sigma0D parameters of GaMD. The average GaMD boost’s potential values in the production simulations were between 13.8 and 15.6 kcal/mol.

Trajectory analysis was performed using CPPTRAJ [38]. For conformational clustering, we used the K-means algorithm. Before clustering, the frames from each trajectory was aligned on the energy-minimized starting structure for each type of system. We used the RMSD between the α-carbon atoms in the two coagulation factors as the distance metric in the clustering. The clusters were numbered by the algorithm according to the number of structures in each, so that the same cluster number in two groups of simulations (e.g., Cluster 1) did not imply similar conformations.

## 3. Results and Discussion

### 3.1. Result of Protein–Protein Docking

To investigate the interactions of FXa and FIXa with the exosite of AT not binding a heparinoid ligand, we have followed two different approaches. First, we built model structures using protein–protein docking with HADDOCK, starting from a conformation of AT with a closed hinge region. Second, we have studied model systems that were constructed directly using the X-ray structures, but with the pentasaccharide deleted.

**Figure 1:**
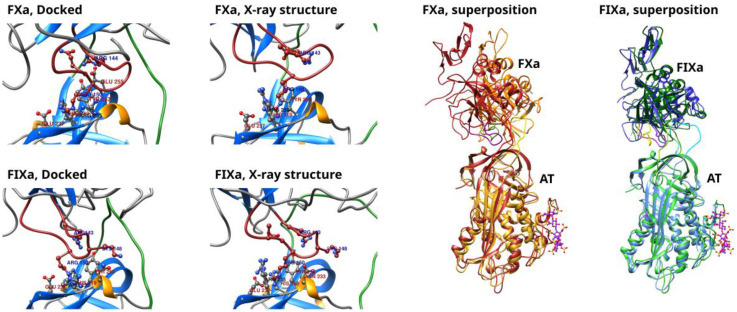
Comparison of the exosite interactions in the AT–FXa and AT–FIXa structure obtained by docking and the corresponding X-ray structure. In the first two columns, the exosite interaction is compared between the docked structure and the corresponding X-ray structure. The autolysis loops of the factors are shown in red and the RCL of AT is colored green The superpositions of the “docked” and “X-ray” conformations are shown in the two columns on the left side. In the superposition, the docked structures are shown in red for the FXa complex and in green for the FIXa complex. The X-ray based structures are colored yellow and blue. The 148-loop has a different color than the rest of the FXa or FIXa protein. The pentasaccharide ligand, present only in the X-ray structures, is shown in purple.

HADDOCK was able to build structures for both factors with the “hinge” region in its closed state. In the structures selected for molecular dynamics simulations—the structure with the highest score in the highest-ranked conformational cluster—all three amino acid contacts defined as “unambiguous restraints” were present (see the Methods section for further details.).That is, the Arg393–Ser394 peptide bond and the Arg393 side chain were in the proper position for the enzyme reaction and the critical Arg150 side chain [9] was also interacting with the surface of the exosite. The closed “hinge” conformation has an effect on the conformation of the entire RCL. For optimal interactions with both the RCL and the exosite of AT at the same time, the autolysis (or 148-) loop in the factors must undergo conformational adaptation. The position of this loop, as well as the exosite interactions in the docked and the corresponding X-ray structures are compared in Figure 1 for both factors. In the two structures obtained by docking, the Arg150 side chain interacted with the same region on the surface of AT, close to residues Tyr253 and His317; this is expected as this interaction was defined as a restraint in docking. In both factors, the autolysis loop contained two additional positively charged residues, Arg143 and Lys148, which were also involved in the interaction with the exosite [12]. In the X-ray structure of the AT–FXa complex, the Arg143 residue interacted with Glu255 in AT. Arg143 was oriented towards Gln255 in the corresponding docked structure also, but the distance between the residues was higher. The Lys148 side chain was missing in the X-ray structure, and this made the comparison difficult for this amino acid. In the HADDOCK-generated structure, the same residue was oriented towards the RCL of AT. As for the FIXa complex, both residues interacted with the Glu255 of AT in the experimental structure, but in the selected conformation form of the docking, Arg143 was not close enough to the Glu due to the different position of the 148-loop.

### 3.2. Interaction between the Arg150 Residue and the Exosite of AT in the Docking-Based Simulations

**Figure 2:**
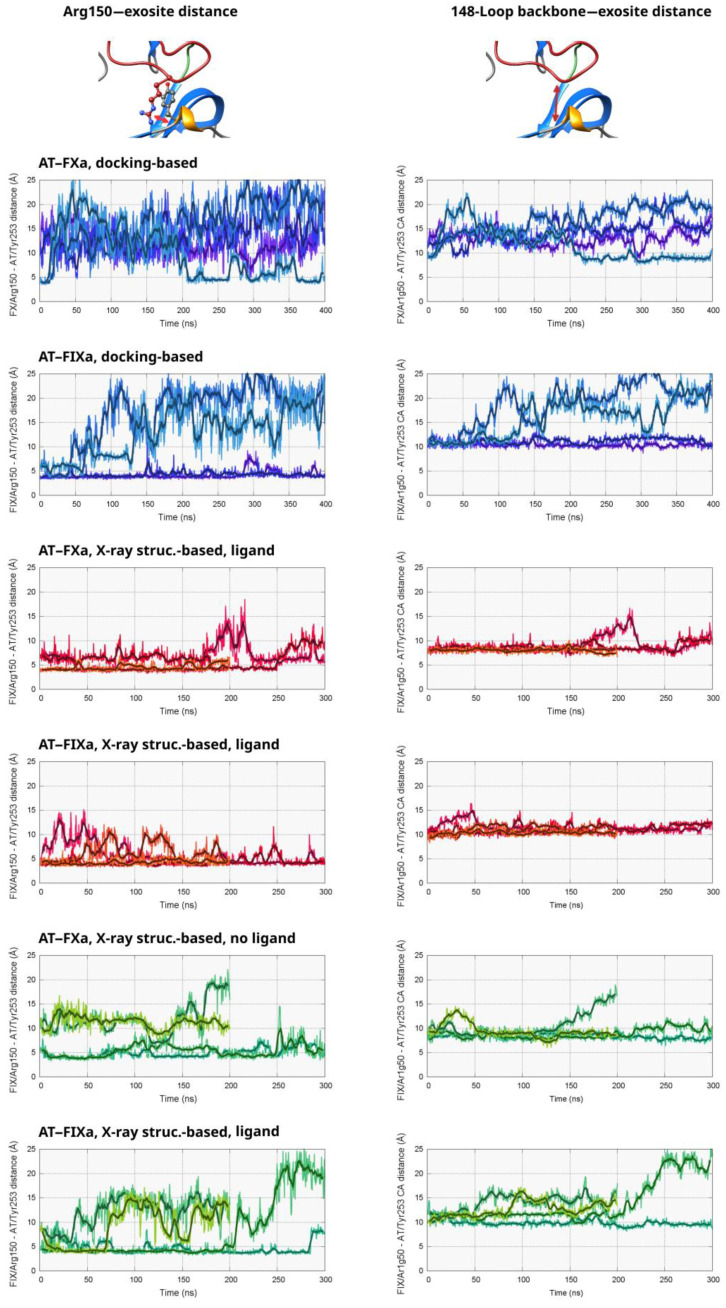
Interaction of the Arg150 residue of FXa and FIXa with the “exosite” of AT. For each “group” of simulations, the distance between the CZ atom (in the guanidino group) of the Arg150 residue and the Tyr253 in the exosite of AT is shown as a function of time in the left column of the figure. For comparison, the distance between the peptide’s backbone α-carbon atoms between the same two amino acids are also shown in the right column.

The critical role of the autolysis loop (or 148-loop) of both FXa and FIXa in the interaction with AT is supported by both X-ray diffraction structures [9,10] and mutagenesis studies [11,12,39]. The Arg150 amino acid in this loop is especially important as it forms direct contacts with the surface of the serpin near the Tyr253 amino acid. To study the binding of this critical residue to AT in the non-pentasaccharide binding state, we calculated the distance between the side chain of Arg150 (CZ atom) and the β-carbon of Tyr253 in AT from the simulations that started from our docked structures. The distance values are shown as a function of the simulation time in Figure 2 and in Figures S1 and S2.

The results for the four simulations started from the docked AT–FXa complex are shown in the top left part of Figure 2. It is evident from the distance values that the Arg150 did not bind very tightly to its binding site found in the X-ray structure. The “combined” conformational space explored by the four copies of the system was very large, with distances as low as 5 Å and as high as 22 Å, as well as many “intermediate” conformations. In one of the simulations (Number 4 in Figure S1), the system exhibited a different behavior compared to the other three. The Arg150 residue started unbinding from its binding site on AT very soon and reached distances above 20 ns after about ~80 ns. The sidechain bound again into its pocket in the AT exosite at ~210 ns, and the distance value remained below 10 Å in most of the remaining part of the trajectory. We were not able to capture any similar event in the other simulations of the system. However, this observation at least indicates that the transition between states exhibiting a “strong” or “weak” Arg150 interaction were reversible and that the energy barrier was low.

The conformational behavior was very different in the four simulations based on the docked FIXa structure. In two trajectories, very limited movement of the Arg150 residue can be observed out of its binding “pocket”, demonstrated by the distance values below 5 Å. In the remaining two simulations, this amino acid unbound from the serpin surface between ca. 80–150 ns. With the exception of relatively short periods, the distance of Arg150 remained high from the exosite in the rest of the two trajectories.

To investigate the position of the autolysis loop without the conformational fluctuations of the Arg150 sidechain, we also analyzed the distance between the peptide backbone near Arg150 in the two factors and the AT exosite (right column of Figure 2, Figures S3 and S4). In AT, the α-carbon atom of Tyr253 was selected for the analysis. Typically, there is a strong correlation between this “backbone” distance and the value for the Arg150 side chain. However, the lowest and the highest observed values are much closer to each other in the former case than in the latter, indicating that the 148-loop itself moved less than did the Arg150 side chain.

To analyze the distribution of the “exosite” distance, we also plotted the “backbone distance” parameter in histograms (Figures S4 and S5). In the two AT–FIXa simulations showing large conformational changes, a region around 12–15 Å was visible with low frequencies, suggesting that these conformations are less favorable.

### 3.3. The Allosteric Effects of Pentasaccharide Binding on the Exosite Interactions in the X-ray Structure-Based Simulations

Several papers have been published in which the allosteric effects of ligand binding to AT were studied based on MD trajectories for the protein [18,20,40,41]. In contrast, simulating the Michaelis complexes of AT with FXa and FIXa allowed us to study these effects directly. Therefore, we performed simulations based on the X-ray structure of the Michaelis complexes with or without a pentasaccharide to gain insights into the allosteric activation mechanism. We performed four independent simulations for all four systems (FXa vs. FIXa complex, and pentasaccharide vs. no pentasaccharide), with two 300 ns and two 200 ns runs in each case.

To investigate the interaction of Arg150 with AT, we analyzed the same atom distances as those in the docking-based simulations. As expected, conformations with a larger Arg150 distance from the exosite were observed to have a much lower frequency than that in the docking-based trajectories. However, it was still possible to draw conclusions on the effects of the pentasaccharide binding from the results. The results are shown in Figure 2 (the “red” and the “green” plots) and in Figures S1–S4.

In two simulations of the FXa system with a pentasaccharide (3 and 4 in Figure S1), the Arg150 side chain of FXa was in a position very close to its binding site in the X-ray structure with only very small fluctuations. In the other two trajectories, we could observe somewhat larger variations in this parameter over the simulation time, but the distance was still well below 10 Å in a large majority of the sampled states. In the simulations not containing a pentasaccharide, the results were not significantly different from the ligand-containing ones. However, in runs 3 and 4, the interaction between the Arg150 sidechain and its binding site was less tight. The distance was above 10 Å by more than half in both trajectories, and values close to 20 Å could be observed close to the end of Simulation 3.

Regarding the simulations based on the AT–FIXa X-ray structure, the tendencies were largely similar. In the simulations with a pentasaccharide, the Arg150 remained close to Tyr253 in the case of most conformations, with some level of flexibility in its position in Simulation 1 and 3. In contrast, a significant number of states with distance values close to or above 15 Å were present in three of the four trajectories lacking the AT ligand, and such states were almost completely absent from the FIXa simulations with the heparinoid.

Similarly to the docking-based simulations, we also investigated the distance between the peptide backbones of the interacting protein pairs at amino acid 150 in the factor and Tyr253 in AT (right column of Figure 2). As expected, the “backbone distance” fluctuated much less than did the values for the Arg150 side chain. In agreement with the results from the docking-based MD, significant movements of the Arg150 side chain were observed in those simulations which also showed an increase in the backbone distance. Due to the relatively short simulation times, we could not observe larger conformational changes in AT towards the non-activated state after the deletion of the pentasaccharide. However, in the GaMD simulations based on the X-ray structures, we were able to observe signs that the presence (or the absence) of the pentasaccharide had an allosteric effect on the interaction of Arg150 with the exosite of AT.

We also analyzed the “backbone distance” parameter through histograms (Figures S5 and S6). In the FIXa complex system, conformations with a distance value above 10 Å occurred in the simulations not containing a ligand. The tendencies were similar in the AT–FXa simulations; however, the difference was smaller due to the somewhat higher conformational variability in the pentasaccharide-containing state compared to the AT–FIXa complex.

However, the conformational changes described above were not observed in all simulations of the same type of system. Therefore, more conformational sampling would be needed to better understand these processes.

From the simulations containing a pentasaccharide, we have also assessed the binding of the ligand using the same “RMSD” metric as that used in our previous papers [20,22] (Figure S7). The ligand binding to AT in the pentasaccharide-containing systems was stable, but there was some level of conformational variability according to the RMSD analysis. Apparently, these small movements of the pentasaccharide did not correlate with the changes in the interaction with the exosite.

### 3.4. Analysis of Interactions between Amino Acid Pairs

**Figure 3:**
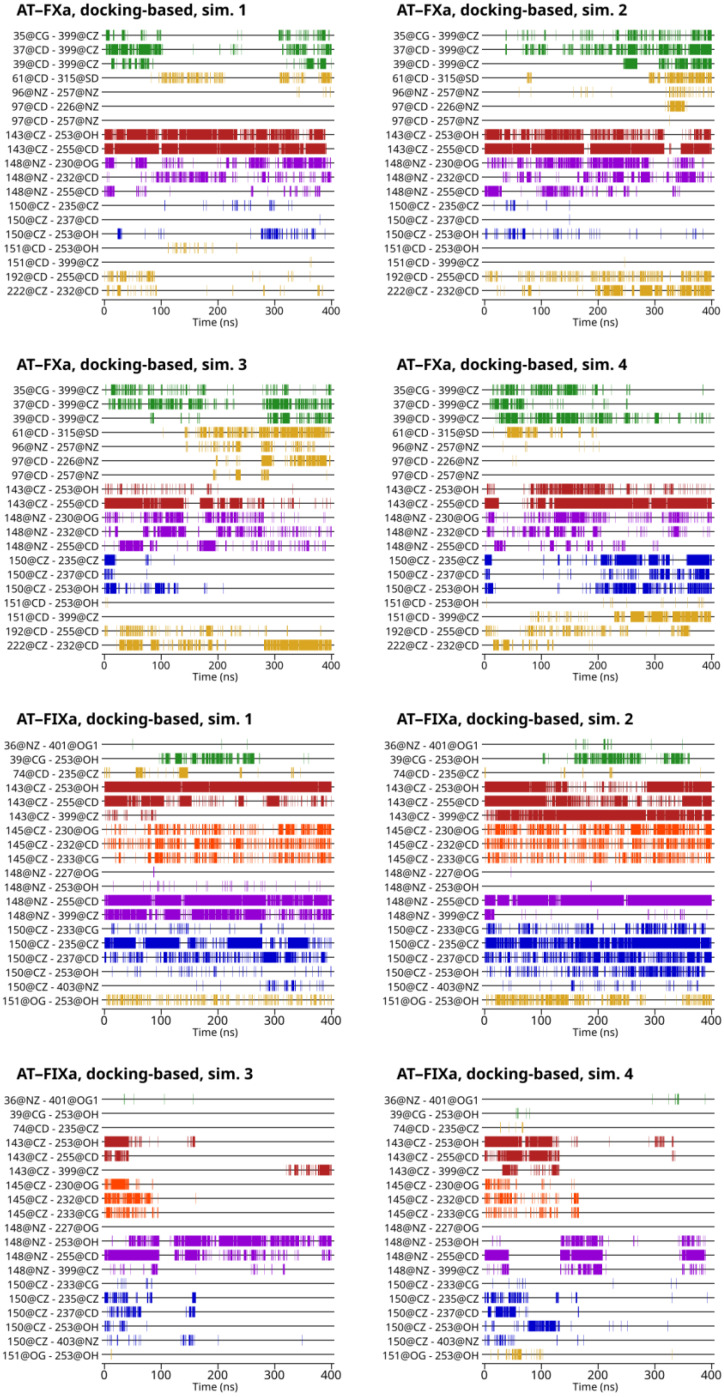
The amino acids involved in exosite interactions, as a function of time, in the docking-based simulations. The first number is the residue in factor Xa or IXa, while the second is the amino acid in AT. A residue pair is shown as “interacting” if the distance is below a cut-off value of 6 Å. Different colors are used for each residue in FXa or FIXa.

For understanding the differences in the exosite interaction between the simulated systems—that is, the two factors and the different activation states of AT—it is essential to know which amino acid pairs were involved in the interaction. The interacting pairs included in the analysis were selected using the representative conformations from the cluster analysis of all six groups of simulations. The results are shown in Figure 3 for the docking-based trajectories and on Figures S8 and S9 for the X-ray diffraction-based trajectories. We defined a residue pair as “interacting” if the distance was below a cut-off value of 6 Å.

In Figure 3, a conformational change in two simulations based on the FIXa-docked structure is visible. After ~100 ns in Simulation 3 and ~180 ns in Simulation 4, very few interactions remained between the two proteins. A particularly interesting example is Arg143. This residue interacted with Glu255 in AT in most simulations discussed in this study (FXa, FIXa, docking and X-ray structure-based). The only exceptions are the two simulations of the FIXa-docked structure that showed significant unbinding of Arg150 from its pocket. The interaction was present at the beginning of both simulations, but the distance increased between the two sidechains in parallel with the partial dissociation of the Arg150 residue, as discussed before. The notable exception is the interaction between Lys148 of FIXa autolysis loop and Tyr253 and Glu255 in AT, as well as a few other contacts in short time intervals in Simulation 4. Some of the very few remaining contacts were even repulsive, being found between positively charged Arg residues. The behavior of the docking-based FXa simulations was markedly different. Even in the conformations with larger distances between the autolysis loop and the AT exosite, the interactions were still present between several amino acid pairs.

According to the available experimental data, the interaction of FIXa with non-activated AT was weak, even relative to the FXa binding to AT in this state. This raises the question of how this can be explained with our MD data. The difference in interaction’s free energy may be due to the combination of two factors with opposite effects. In the docking-based FXa complex simulations, the binding of the Arg150 residue appeared to be less stable to the AT exosite, but this may have been compensated by several other, less specific interactions between the two proteins. In contrast, the simulations of the AT–FIXa systems suggested the existence of a state with favorable binding that could exist for longer time periods, but there was very limited interaction in other conformations. Additionally, the transition was observed in one direction, and this may suggest that the opposite process (from a large distance to close contact) may not have been kinetically favorable.

The data obtained from our simulations are certainly not sufficient for an accurate comparison of the stability of the states discussed above. However, we still expect that the conformations observed in our simulations—not previously reported—provide insights into the biochemical background of the weaker interaction of non-activated AT with the factors.

#### 3.4.1. Contribution of the Arg143 and Lys148 Amino Acids

As mentioned before, the autolysis loop of both factors contains two further amino acids with a positive charge, Arg143 and Lys148. Izagurre et al. have published mutagenesis studies involving these two amino acids in factor Xa [12]. However, to our knowledge, no such data are available for factor IXa.

The Arg143 amino acid of FXa and FIXa mainly interacted with one AT residue in the AT–FXa complex simulations, Glu255. Additionally, it could be in close proximity to the ‘binding pocket-forming’ Tyr253 amino acid. Arg143–Glu255 was one of the most stable interactions in these simulations as it was present in the majority of the sampled states in all 12 simulations. The role of Arg143 was largely similar in the AT–FIXa model systems: its most important interaction was with Glu255 and could be positioned close to Tyr253. However, it could also be found close to Arg399 in some of the states in the trajectory. The Arg143–Glu255 interaction was “stable” except in the two trajectories where a conformational change occurred.

In the AT–FXa complex simulations, the Lys148 in FXa appeared to play a more important role in the docking-based simulations than it did in the X-ray diffraction-based trajectories. This residue could interact with two positively charged residues in AT, Glu232 and Glu255. In the docking-based simulations, both interactions were present in a large number of sampled conformations, while at least one of the two was absent or very weak in the X-ray-based simulations. In the AT–FIXa complex trajectories, Lys148 couldonly interact with Glu255, but not Glu232. The Lys148–Glu255 distance varied significantly between each trajectory and with the simulation time. Notably, Lys148–Glu255 was one of the very few interactions that remained in Simulation 3 after the conformational change to a state with a weaker interaction.

The Gln151 residue of the autolysis loop of FXa could make contact with the Arg399 amino acid of AT. This interaction was almost entirely absent in the docking-based simulations, but was found in a significant number of conformations in the X-ray diffraction-based simulations. Residue 151 was substituted with a serine in Factor IX. This residue was close to the binding site in most of the simulation time, except in the two trajectories where the aforementioned conformational change occurred.

In the autolysis loop of factor IXa, one further amino acid made close contact with AT, namely Phe145. Its side chain was positioned close to Glu232 and Asn233 in AT. This interaction was stable in all simulations of FIXa except the two where the previously discussed conformational change occurred. In those simulations, the interaction disappeared in parallel with the unbinding of Arg150, Arg143 and other residues.

In FXa, two further residues could be found in contact with the surface of AT, Gln192 and Arg222. Such conformations were mainly found in the docking-based trajectories and were almost completely absent from the simulations containing a pentasaccharide ligand.

#### 3.4.2. Involvement of Regions Outside the Autolysis Loop in the Exosite Interactions

Yang et al. have suggested that the sequence differences in the 36-loop (or 39-loop) between FXa and FIXa may be partially responsible for the slower reactivity of FIXa with non-activated AT [42]. Notably, this structural element contains three negatively charged Glu residues in FXa (residues 36, 37 and 39), while in FIXa, residue 37 is missing and the other two are substituted with a Lys and an Asp, respectively.

As expected, the main interaction of all three Glu residues was with the Arg399 residue in AT (Figure 3). Such interactions were present in all three types of systems investigated (docking-based and X-ray diffraction-based, with and without a pentasaccharide). In some trajectories, however, such binding was limited to specific parts of the trajectory. This suggests that this interaction depends on the binding mode of FX. From our results, it is clear that the 36-loop of FIXa could not interact with Arg399 in the AT in any of the three states simulated. Instead, in two of the four docking-based simulations, the Asp39 residue was frequently found in proximity to the Tyr253 residue of the binding site in AT. This was not observed in any of the simulations based on the X-ray diffraction structure. Residues Gln61, Lys96 and Glu97 in factor X were also observed to make contact with AT. Although Gln61 was located in a different loop than the other two, it seems reasonable to discuss them as a single group. These amino acids were located far from the surface of AT in the X-ray structure. However, there were some interactions between these amino acids and AT in all four docking-based simulations at least in some part of the trajectory. This interaction was absent from the X-ray diffraction-based simulations, with the exception of one simulation without a pentasaccharide.

### 3.5. Cluster Analysis

**Figure 4:**
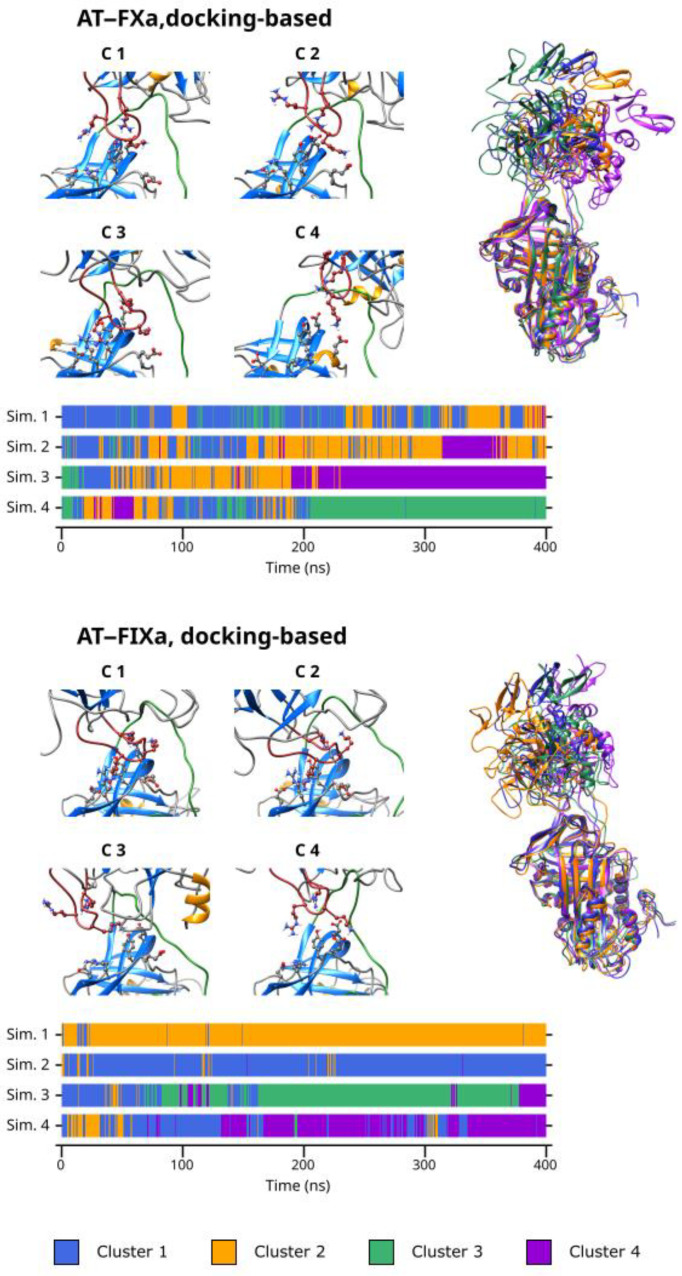
The result of cluster analysis for the docking-based simulations. The figure consists of three parts for both complexes: 1. the 148-loop (red) and the exosite in the representative conformations, 2. the superposition of the representative conformations on the right, and 3. the cluster number as a function of time, below the representative frames. In the superposition figures, AT and the coagulation factor are shown in the same orientation as they are in Figure 1.

As discussed before, our simulations revealed that multiple binding modes exist in both the AT–FXa and the AT–FIXa complexes. We performed cluster analysis to identify conformational types in both the docking- and the X-ray diffraction-based simulations. The cluster number parameter for the K-means algorithm was four in the docking-based systems but only three in the X-ray diffraction-based simulations as these have smaller variability in conformation. The results obtained from cluster analysis of the docking-based simulations are presented in Figure 4. The figure shows the representative conformation for each cluster as well as the cluster number as a function of time. The superpositions of the conformational clusters are found in the right column, with each structure colored according to the cluster number.

From the result of the cluster analysis, it is evident that the four docking-based simulations of the FXa system together explored a large conformational space. Cluster 3 and 4 represent the two extremes of the “spectrum” of conformations observed, while Cluster 1 and 2 are “intermediate” states between them. Cluster 3 contained the conformations with the most favorable binding mode of Arg150, while the distance between the autolysis loop of FX and AT was the highest in Cluster 4. Most of Simulation 1 and 2 as well as the first ~200 ns of Simulation 3 and 4 are characterized by frequent and rapid changes between the conformational types. However, in the second half of the latter two simulations, most conformations belonged to a single cluster—Cluster 4 in the former and Cluster 3 in the latter trajectory. This, of course correlates with the large (in Simulation 3) and small (in simulation 4) Arg150exosite distances observed in the simulations.

**Figure 5:**
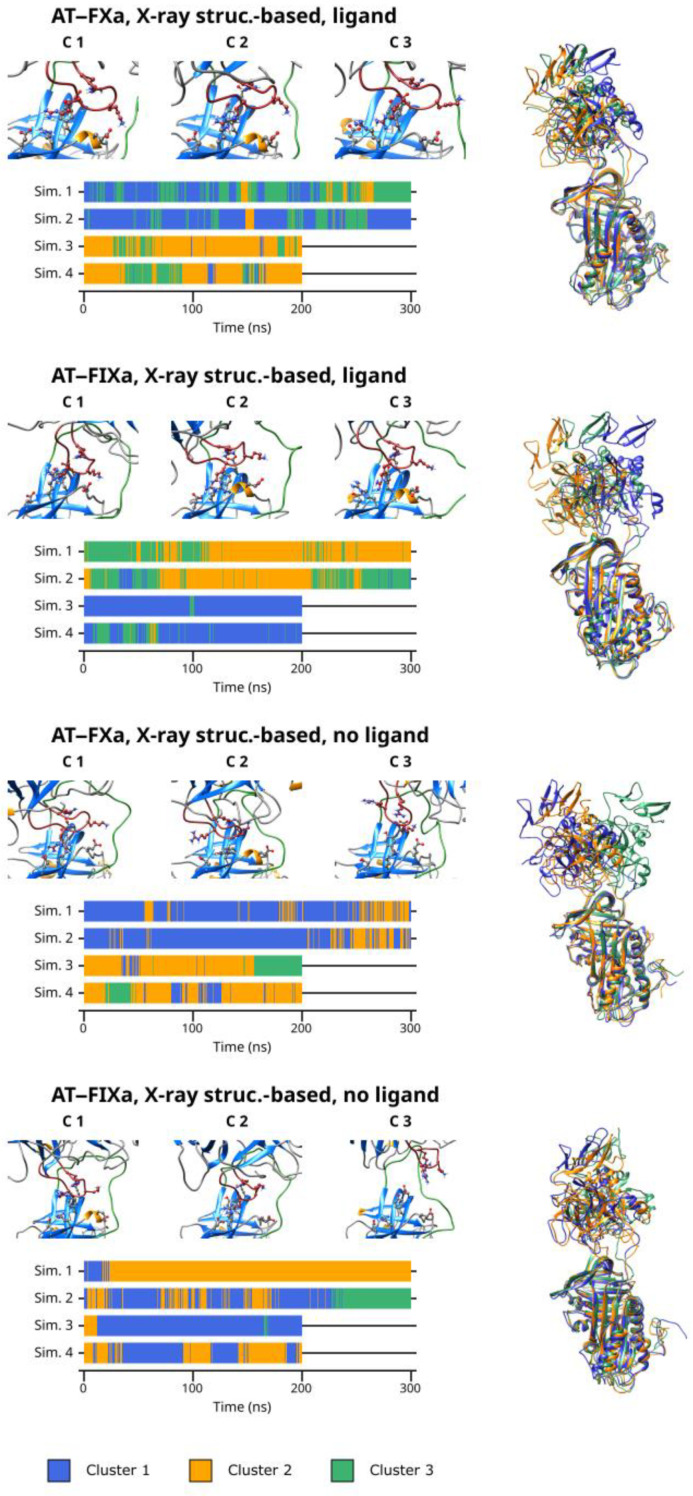
The result of cluster analysis for the X-ray diffraction-based simulations. The figure consists of three parts for both complexes: 1. the 148-loop (red) and the exosite in the representative conformations, 2. the superposition of the representative conformations on the right, and 3. the cluster number as a function of time, below the representative frames. In the superposition figures, AT and the coagulation factor are shown in the same orientation as they are in Figure 1, Figure 3 and Figure 6. Root mean square fluctuations.

Similarly, the docking-based FIXa system had significant conformational flexibility. However, a few important differences are evident (Figure 5). Here, Cluster 1 and 2 contained the conformations with a more-or-less optimal interaction between the autolysis loop and the AT exosite, while the states showing a less tight interaction were placed in the remaining two clusters. At the beginning of Simulation 3 and 4, the sampled conformations belonged to Cluster 1 and to a lesser extent, Cluster 2. This corresponds to the states in which a short distance was observed between the Arg150 residue and the exosite. The previously discussed conformational change in the other two simulations are clearly visible in the cluster analysis results. After this event, Cluster 3 became ‘dominant’ in Simulation 3 and Cluster 4 became dominant in Simulation 4.

Even in the X-ray diffraction-based simulations with a pentasaccharide, the position of the factor compared to that of the AT differed significantly between the three clusters. This demonstrates the remarkable flexibility of the system despite the stable binding of the Arg150 residue in a large majority of the conformations analyzed. Apparently, the main cause of this conformational variability was the flexibility of the RCL but the position of the 148-loop could adapt to the changes.

The cluster analysis of the X-ray structure-based simulations without the ligand revealed both significant similarities and differences between the two factors. In both cases, the first two clusters represent the movements of the proteins within the complexes in states with close exosite interaction, while the states with an apparently less favorable binding modes were placed in the third cluster. When comparing the Cluster 3 conformations obtained for the FXa and the FIXa systems, the large difference in the position of the factors is clearly visible. This further supports our conclusions about the different binding and unbinding mechanisms between the two factors.

In general, the cluster analysis revealed multiple important aspects of the interaction. First, it demonstrated the highly dynamic nature of the interaction, even in simulations where Arg150 was in stable interaction with the AT exosite. Second, it provided further insights into the differences in the conformational space available to the two factors.

Root mean square fluctuations (RMSF) are a simple and frequently used technique for measuring the flexibility of specific parts of a protein from an MD trajectory. In the present study, we performed the calculation separately for AT and the clotting factor Xa or IXa for all simulations. The RMSF values from the individual simulations are plotted in Figures S10–S13. For all α-carbon atoms, averages were calculated from the four simulations for all six systems (docking-based and X-ray structure-based, the latter with or without a pentasaccharide) for both factors. In each of the four plots, we compared the averaged fluctuations for the three types of model systems.

**Figure 6:**
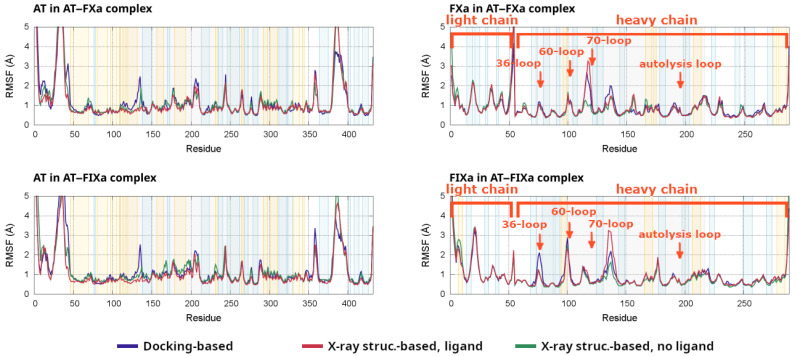
The result of the RMSF analysis for the α-carbon atoms. For both factors, the results for AT are shown on the left and the results for the factors are shown on the right. The residue numbers for the factors include both the light and the heavy chain and differ from the chymotrypsin numbering used elsewhere in this article. The plotted RMSF values are averages from the four simulations belonging to each group.

Results from the analysis are presented in Figure 6 for both AT–factor complexes studied. In both cases, fluctuations in AT are shown on the left and the RMSF values in the clotting factor Xa or IXa are shown on the right. Due to technical reasons, the X axis for FXa and FIXa uses a different numbering than that used in the rest of the paper. Instead, we added labels for the most important loops in these two plots, showing the residue numbers used in the text.

The RMSF analysis of AT provided insights into the consequences of the pentasaccharide binding on the conformational behavior of the system. In several regions of AT, the fluctuations were the smallest in the X-ray-based simulation with the pentasaccharide. In the region close to the heparin binding site (100–140), however, significantly higher fluctuations in the values could be observed in the docking-based systems compared to the values from the X-ray diffraction-based simulations of both factors. This indicates that the conformation of several structural elements in the region were stabilized once all conformational changes triggered by the pentasaccharide had taken place. The difference was the largest close to the C-terminal end of helix D, which underwent a conformational change in the later steps of activation.

The most notable difference between the FXa and FIXa complexes is that the removal of the pentasaccharide caused much higher increases in the fluctuations of several regions in the simulations of FIXa. These increases were most visible between amino acids 80–200 in AT, and this region includes much of the heparin binding site, two strands off beta sheet A as well as helices E and F. This may indicate that the conformation of AT was more sensitive to the pentasaccharide binding in the FIXa-containing simulations, compared to that in the FXa-containing ones.

Of particular interest is the region around amino acids 230–240 in AT, which includedmultiple residues involved in the exosite interaction. The differences were not particularly significant in this region among the groups of simulations analyzed. However, the pentasaccharide-containing simulations tended to have lower values for both factors. The lower fluctuations may indicate a conformation more favorable for interaction with the factors. It should be noted, however, that this was a region that underwent only smaller visible conformational changes.

As expected, the differences between the activation states had relatively small effects on the RMSF values in the catalytic domains of the factors. However, we could observe important differences between FXa and FIXa. The 36-loop, as mentioned before, contributed to the AT–FXa interactions, but its role was less important in the AT–FIXa complex. Interestingly, the RMSF of this loop was higher in the docking-based simulations of the AT–FIXa complex than it was in any other systems. This corresponded to a conformational change that resulted in the Asp39 residue becoming better-positioned to interact with the exosite. The flexibility of the 60-loop was not significantly higher than that of other loops in FXa. In contrast, in the FIXa complex simulations, it was one of the most flexible parts of the heavy chain. The behavior of this region was similar between the docking-based and X-ray based simulations, with slightly higher RMSF values in the former group. In contrast, the 70-loop or the calcium binding loop was found to have a higher tendency to conformational changes in the FXa simulations based on the RMSF values. Interestingly, the autolysis loop did not show significantly higher fluctuations than those of several other loops in the factor, despite our observation of some level of conformational flexibility. This may indicate that the conformation of this structural element was stable to a limited extent and that this is required for interaction. The RMSF values were somewhat higher in the docking-based simulations of the two factors compared to those in the X-ray diffraction-based ones. This may correspond to the “conformational adaptation” of this loop to the changes in the relative position between the proteins.

### 3.6. Conformation of the Hinge Region

**Figure 7:**
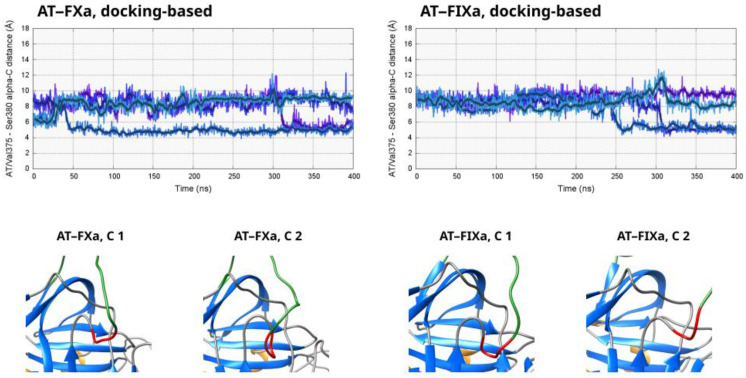
The distance between the α-carbon atoms of AT residues Val375 and Ser380 in the docking-based simulations of both complexes as a function of time. Lower values correspond to “inserted” conformations of the hinge region. To illustrate the possible conformations of this structural element, its conformation in the first two clusters from the docking-based simulations is depicted in the bottom half of the figure. The hinge region of AT is colored red while the rest of the RCL is shown in green. The coagulation factors are not visible.

The N-terminal part of the reactive center loop of AT, often referred to as the “hinge” region, is inserted into β-sheet “A” in the non-activated state [4,5,27]. The expulsion of this region from the β-sheet is one step of conformational changes triggered by pentasaccharide binding [4,6]. This conformational change can have a significant effect on the flexibility of the RCL. One of the main aims of our research was to determine whether or not exosite interaction is possible in “hinge” region conformations other than those found in the X-ray structure. We used the distance between the α-carbon atoms of AT residues Val375 and Ser380 to assess the position of this structural element in the trajectories. The results are shown in Figure 7 for the docking-based systems and in Figures S14 and S15 for all simulations.

In the “preferred” conformation for both docking-based systems, the distance value was 8–9 Å, which is higher than that in a fully closed conformation. This suggests that the AT–factor interaction affected the position of the “hinge” region. The “open” state may have been somewhat preferred in this condition in comparison to one with non-complexed AT. However, another state with distance values of ~5–6 Å and therefore one that was closer to a closed conformation could also be observed in two of the four trajectories in both cases. A conformational transition was also observed form the 8 Å conformations toward the 6 Å ones in both systems, indicating that this change may have been reversible. However, the conformation of the hinge region at a ~8 Å distance was still clearly different from the position of the same amino acids in the X-ray diffraction structures.

### 3.7. Interactions between the RCL of AT and the Clotting Factors FXa and FIXa

The Arg393 residue in the RCL of AT plays a critical role in the process of recognition between the protein and its target factors. The factors contain a binding pocket for this arginine, where its side chain can interact with Asp189 in the factor [9,10]. Otherwise, the amino acid sequence of the RCL is not particularly specific for the binding of FXa or FIXa [11,39]. As a consequence, the interaction with the exosite is also necessary for optimal interactions especially in the activated state of AT. We analyzed the distance between Arg393 and the Asp189 residue of the factors as a function of time. The results are shown in Figures S16 and S17. In most systems, the Arg393–Asp189 interaction was very stable with distance values close to 4 Å. In the docking-based complexes of AT–FXa, however, we could observe somewhat higher values (up to 6 Å). This was probably related to the large movements of FXa relative to the AT in these simulations. In three of the four trajectories, the distance between the two amino acids tended to stabilize around 4 Å in the second half of the simulation time.

In the representative conformations obtained from clustering, we analyzed the amino acid pairs within 6 Å between the RCL of AT and the two factors. The distances were calculated for the α-carbon atoms. The results are summarized in Table S2. The two most important regions of the factors involved were 191–195 and 214–219. A third region, 35–38 also participated in interactions in the factor IXa complexes. The role of this region was different in the AT–FX complex where only residue 35 made contact with the RCL. As discussed before, the role of amino acids 36–39 of FXa in the complex was in binding to the Arg399 site of AT. The nearby Phe41 amino acid of both factors also made close contact with the RCL of AT;in the AT–FIXa complex it was closest to Pro397, while in AT–FIXa it interacted with residues Ser394 and/or Leu395.

### 3.8. Correlation Analysis

The effects of the pentasaccharide on the FXa and FIXa inactivation of AT can be explained mainly by the conformational activation mechanism. The allosteric pathways behind this process can be studied from MD trajectories by analyzing the “correlated motions” between the backbone atoms. Our assumption was that analysis of the allosteric processes from simulations of Michaelis complexes can reveal new details of the mechanism in addition to the previous MD-based studies of AT. For the analysis of correlated motions, we calculated dynamic cross correlation matrices (DCCM) for the α-carbon atoms of AT in all simulations. The results are shown in Figures S18 and S19 for the two factors.

The DCCM analysis revealed that the region containing the RCL of the AT (380–400) tended to show correlations with the opposite sign in contrast to many other parts of the protein, especially in the simulations with the pentasaccharide ligand. This was also visible in the simulations of the X-ray diffraction-based systems without the pentasaccharide, but the absolute values of the correlations were generally lower. This tendency was generally absent from the simulations of the docking-based systems. The results demonstrate that the pentasaccharide binding had significant effects on the conformation of RCL. A similar tendency could be observed in region 150–220, which was visible mainly in the X-ray diffraction-based simulations containing the ligand. The same tendencies could be also observed in the AT–FIX complexes, but they were somewhat less visible. However, the significant differences between the DCCM matrices for simulations of the same starting structure complicated the identification of further regions involved in allosteric pathways.

## 4. Conclusions

The exosite interaction in the AT–FXa and AT–FIXa complexes has been studied using both X-ray diffraction [9,10] and mutagenesis [11,12]. However, experimental structure was only available for the fully activated conformation of AT for both factors. We have performed protein docking and enhanced-sampling molecular dynamics simulations to gain structural insights into the states for which no atomic-level structure has been published.

Using molecular docking, we were able to build complexes for both systems in which the factor interacted favorably with both the RCL and the exosite of AT. This was demonstrated by the binding of Arg393 of AT and Arg150 of the factors. Our simulations were able to provide atomic-level models for states for which no X-ray diffraction structure was available. A further advantage of such simulations is that they can capture the highly dynamic nature of these protein–protein interactions, unlike X-ray diffraction studies can. Even in the simulations containing a pentasaccharide and a very stable Arg150–exosite interaction, the conformational space accessible to the factor relative to AT was remarkably large.

The simulations based on the X-ray structure provide valuable insights into the role of the pentasaccharide in exosite interaction. In the simulations performed without a pentasaccharide, the systems underwent only limited conformational changes towards the non-activated state. Thus, the results should be understood as the early events of the transition. However, even in this state, we could observe signs that the interaction between Arg150 and the AT exosite become less tight in multiple trajectories. For a more complete picture of AT–pentasaccharide binding, even more detailed modeling would be required; however, such simulations would be computationally far more demanding.

Our docking-based simulations revealed how the factors can interact with the “exosite” in AT conformations with a partially closed “hinge” region. The factor binding, however, had an effect on the position of this region, resulting in conformations with a somewhat higher distance from the beta sheet. The simulations of these systems also suggest that the conformational behavior of the two factors might be different. The Arg150 residue in the docking-based simulation of FXa did not bind very tightly to the exosite; however, the loss of the interaction energy due to this was likely compensated by several other interactions. The picture was markedly different in the simulations of the docked FIXa structure. In two simulations, a conformation showing relatively stable interaction between Arg150 and the exosite was observed in the entire trajectory. In contrast, in the other trajectories, a large conformational change took place in a short time period. After this event, almost no interactions remained between the two proteins. Our data are not sufficient for a comparison of the stabilities or free energies of the conformations obtained. However, we expect that the new insights into the conformational behavior of the systems studied could facilitate the design or interpretation of future mutagenesis experiments.

In summary, the ‘enhanced-sampling’ simulation technique applied in our work provided an atomic level model for understanding the interactions in the AT–FXa and AT–FIXa complexes in different activation states of AT.

## Data Availability

The datasets generated for this study are available upon request to the corresponding author.

## Supplementary Materials

The following supporting information can be downloaded at: https://www.mdpi.com/article/10.3390/biom13050795/s1. Figure S1. Distance between the Arg150 sidechain (FXa) and Tyr253 (AT), as a function of time, in the simulations of the AT–FXa complex. Figure S2. Distance between the Arg150 sidechain (FIXa) and Tyr253 (AT), as a function of time, in the simulations of the AT–FIXa complex. Figure S3. Distance between the peptide chain backbone near Arg150 (FXa) and Tyr253 (AT), as a function of time, in the simulations of the AT–FXa complex. The distances were calculated between the α-carbon atoms. Figure S4. Distance between the peptide chain backbone near Arg150 (FIXa) and Tyr253 (AT), as a function of time, in the simulations of the AT–FIXa complex. The distances were calculated between the α-carbon atoms. Figure S5. Histograms for the Arg150 (FXa)–Tyr253 (AT) distances in the AT–FXa complex simulations, showing the distribution of this parameter. The width of bins is 0.2 Å. Figure S6. Histograms for the Arg150 (FIXa)–Tyr253 (AT) distances in the AT–FIXa complex simulations, showing the distribution of this parameter. The width of bins is 0.2 Å. Figure S7. The RMSD of the pentasaccharide ligand compared to its position in the energy-minimized structure, as a function of time, in the X-ray diffraction-based simulations with a pentasaccharide. Figure S8. The amino acids involved in exosite interactions, as a function of time, in all simulations of the AT–FXa complex. The first number is the residue in FXa, while the second is the amino acid in AT. A residue pair is shown as “interacting” if the distance is below a cut-off value of 6 Å. Figure S9. The amino acids involved in exosite interactions, as a function of time, in all simulations of the AT–FIXa complex. The first number is the residue in FIXa, while the second is the amino acid in AT. A residue pair is shown as “interacting” if the distance is below a cut-off value of 6 Å. Figure S10. RMSF analysis ofthe α-carbon atoms of AT, in the simulations of the AT–FXa complex. Figure S11. RMSF analysis of the α-carbon atoms of FXa, in the simulations of the AT–FXa complex. Figure S12. RMSF analysis of the α-carbon atoms of AT, in the simulations of AT–FIXa complex. Figure S13. RMSF analysis of the α-carbon atoms of FIXa, in the simulations of AT–FIXa complex. Figure S14. The distance between the α-carbon atoms of AT residues Val375 and Ser380 in the simulations of the AT–FXa complex, as a function of time. Lower values correspond to “inserted” conformations of the hinge region. Figure S15. The distance between the α-carbon atoms of AT residues Val375 and Ser380 in the simulations of the AT–FIXa complex, as a function of time. Lower values correspond to “inserted” conformations of the hinge region. Figure S16. Distance between the side chains of Arg393 (AT) and Asp189 (FXa), as a function of time, in the simulations of the AT–FXa complex. Figure S17. Distance between the side chains of Arg393 (AT) and Asp189 (FIXa), as a function of time, in the simulations of the AT–FIXa complex. Figure S18. Dynamic cross-correlation matrices (DCCM) for the simulations of the AT–FXa complex. Figure S19. Dynamic cross-correlation matrices (DCCM) for the simulations of the AT–FIXa complex. Table S1. Summary of the simulations performed. The earliest steps of the MD protocol—minimization, temperature and pressure equilibration—are not shown. Table S2. Interaction of the RCL of AT with the two factors. The distances were calculated for the α-carbon atoms in the representative frames of the cluster analysis. A distance cutoff of 6 Å was used.
