# Supplementary material for: The Interaction of Factor Xa and IXa with Non-Activated Antithrombin in Michaelis Complex: Insights from Enhanced-Sampling Molecular Dynamics Simulations
Source: Biomolecules. 2023 May 6;13(5):795. doi: 10.3390/biom13050795 (PMC10216295; doi:10.3390/biom13050795)
Supplement: Supplementary file 1 [file biomolecules-13-00795-s001.zip › biomolecules-2357925-supplementary.pdf]

Supporting Information

The Interaction of Factor Xa and IXa with Non-Activated Antithrombin in Michaelis Complex: Insights from Enhanced-Sampling Molecular Dynamics Simulations

Gábor Balogh and Zsuzsanna Bereczky *

Division of Clinical Laboratory Science, Department of Laboratory Medicine, Faculty of Medicine, University of Debrecen, 4032 Debrecen, Hungary; balogh.gabor@med.unideb.hu
* Correspondence: zsbereczky@med.unideb.hu; Tel.: +36-52-431-956

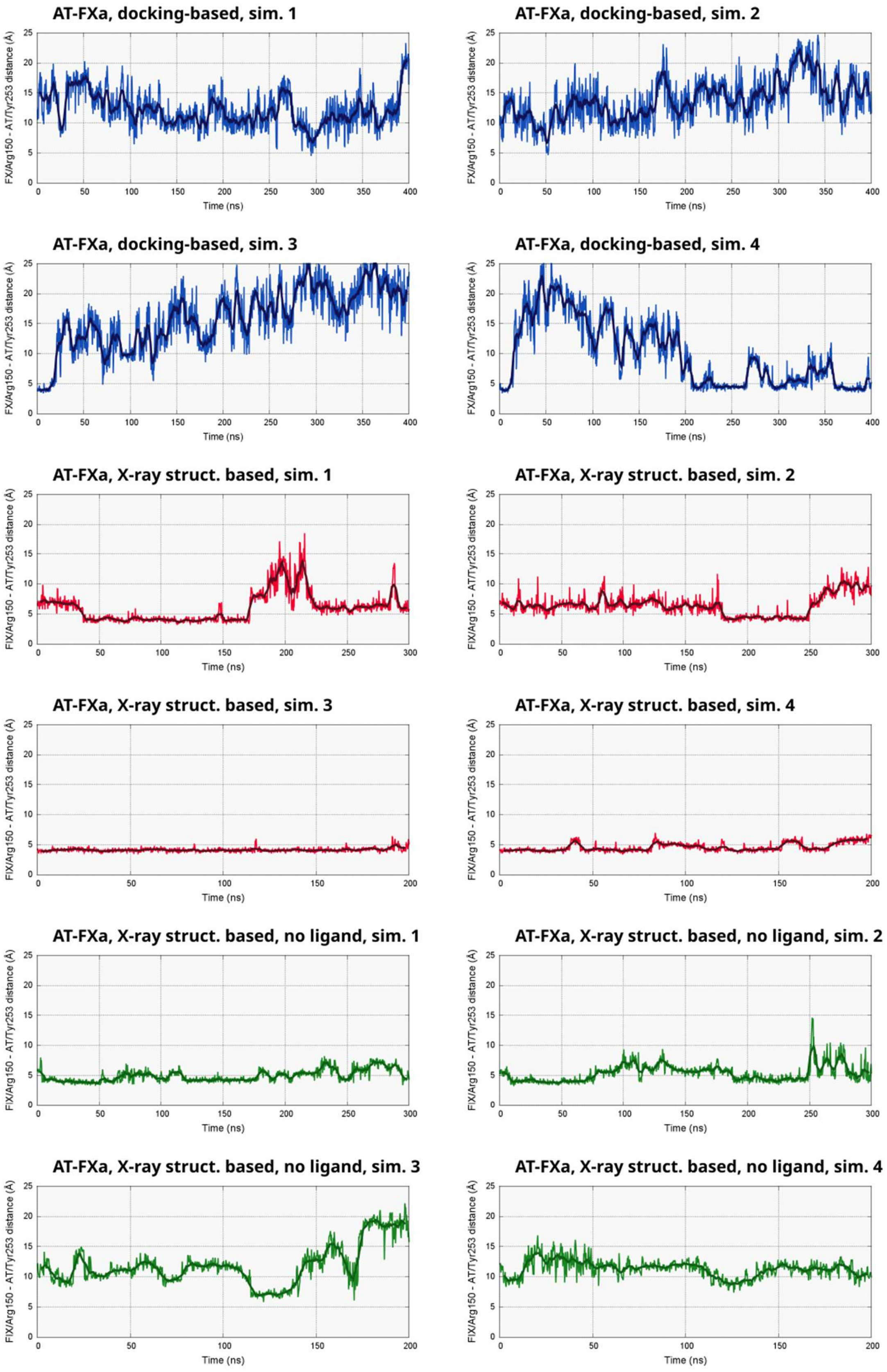

Supplementary Figure S1. . Distance between the Arg150 sidechain (FXa) and Tyr253 (AT), as a func-tion of time, in the simulations of the AT-FXa complex.

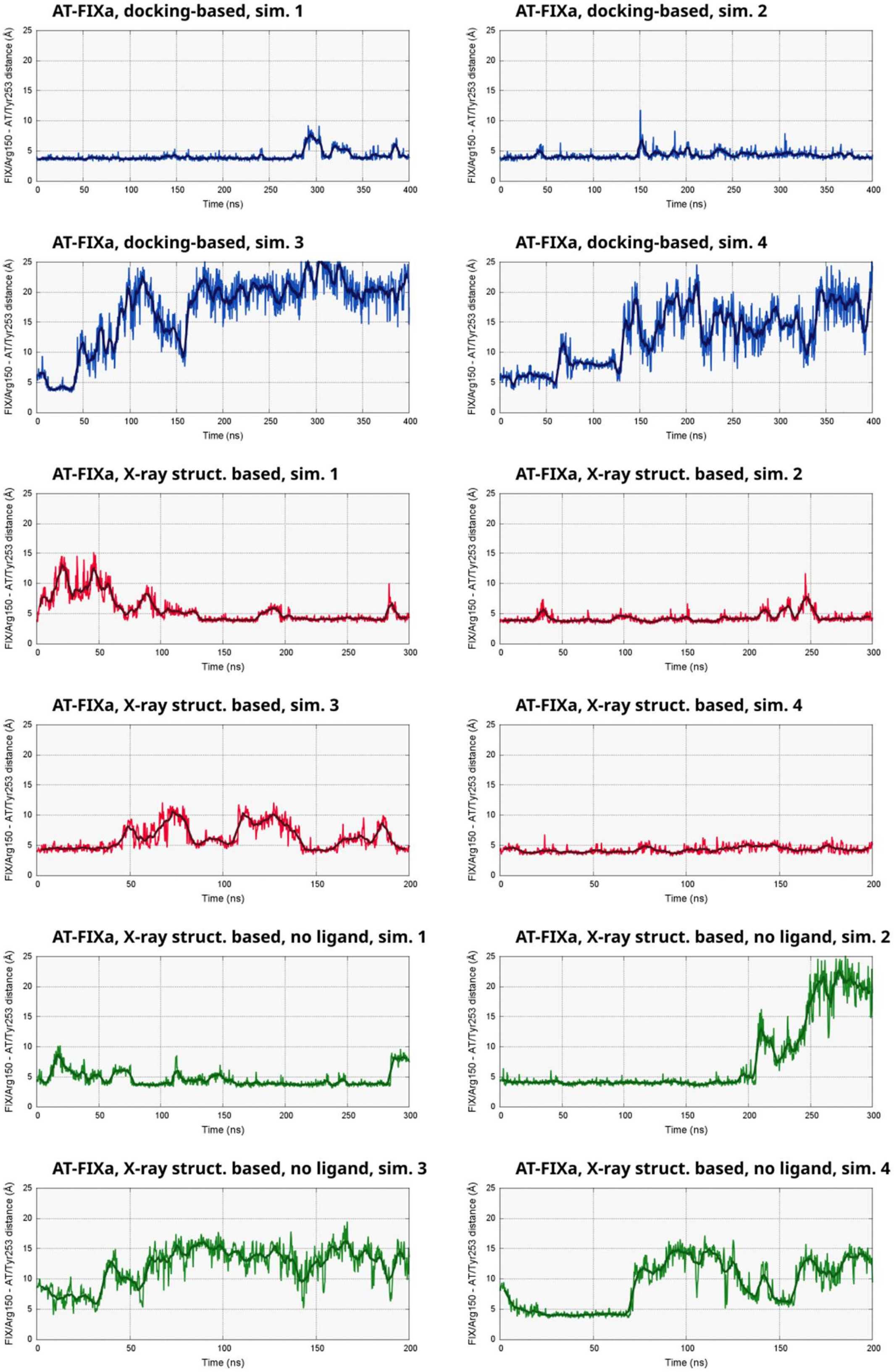

Supplementary Figure S2. Distance between the Arg150 sidechain (FIXa) and Tyr253 (AT), as a func-tion of time, in the simulations of the AT-FIXa complex.

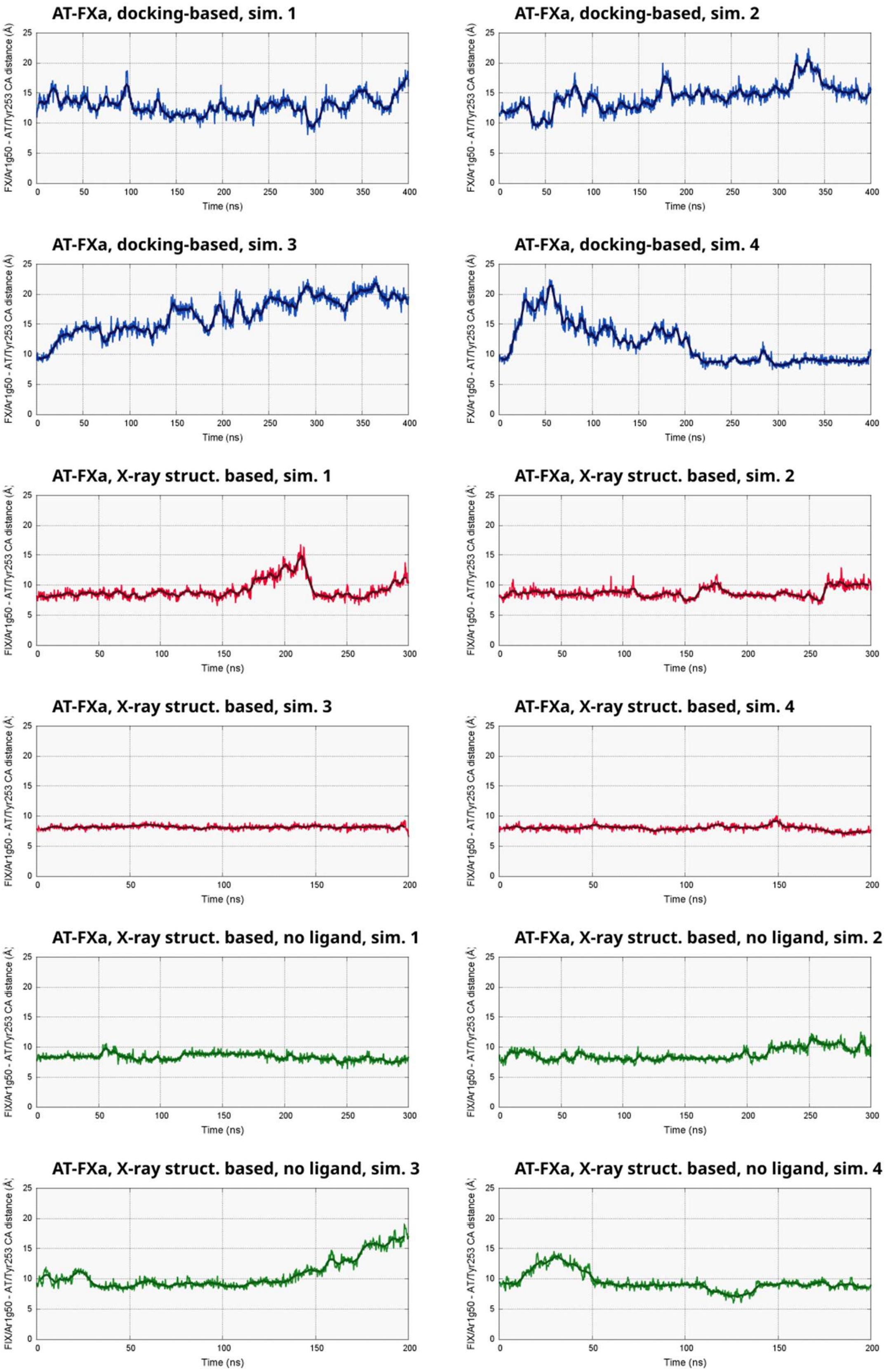

Supplementary Figure S3. Distance between the peptide chain backbone near Arg150 (FXa) and Tyr253 (AT), as a function of time, in the simulations of the AT-FXa complex. The distances were calculated between the α-carbon atoms.

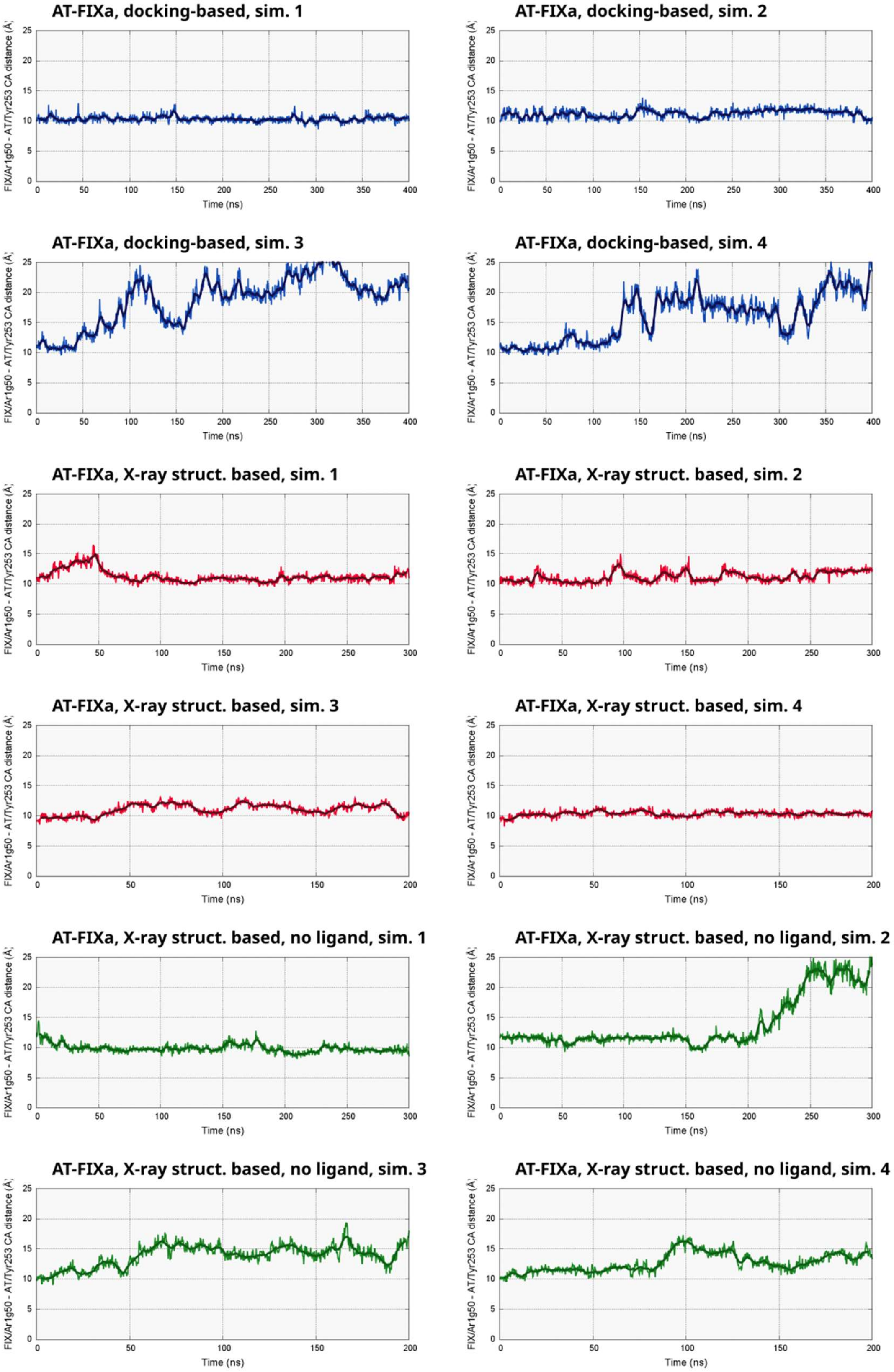

Supplementary Figure S4 Distance between the peptide chain backbone near Arg150 (FIXa) and Tyr253 (AT), as a function of time, in the simulations of the AT-FIXa complex. The distances were calculated between the α-carbon atoms.

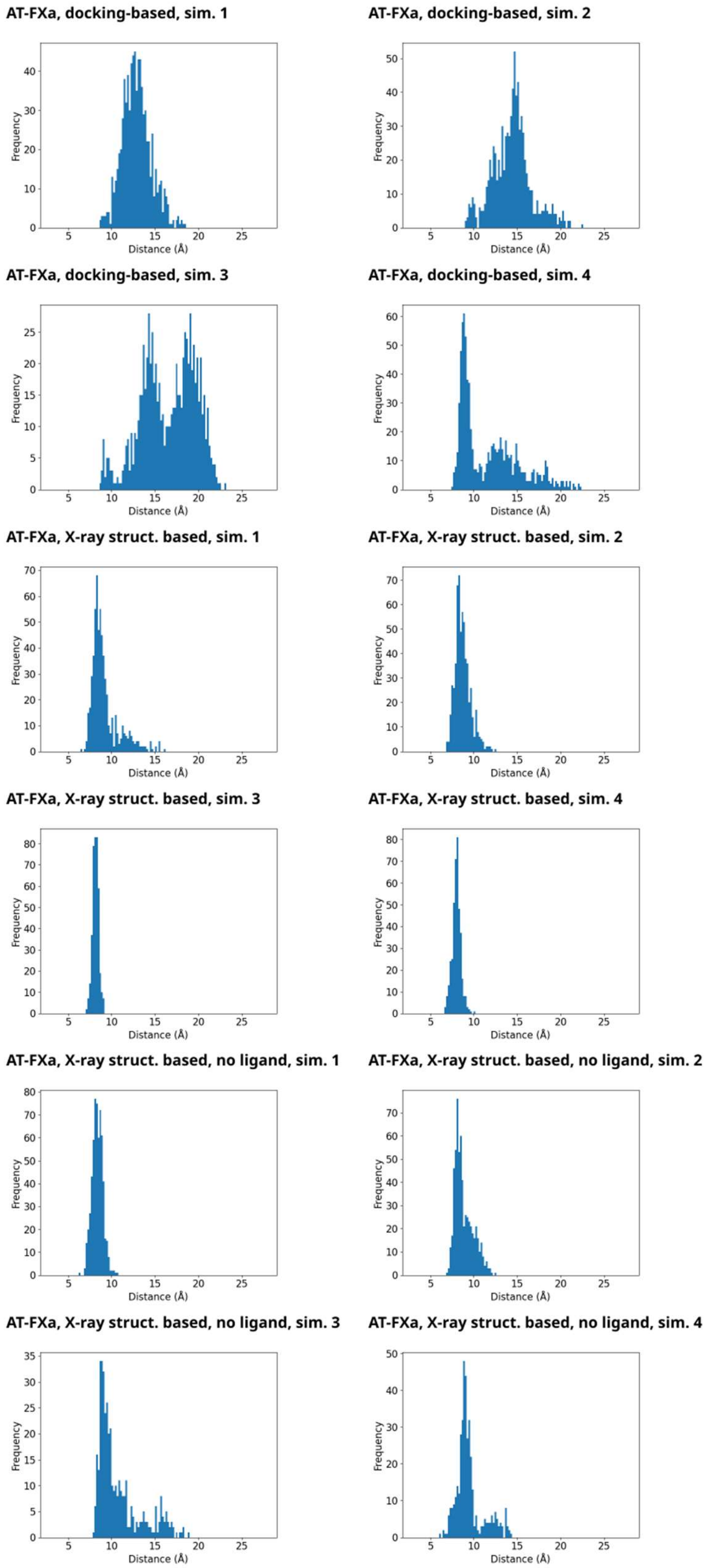

Supplementary Figure S5. Histograms for the Arg150 (FXa) – Tyr253 (AT) distances in the AT–FXa complex simulations, showing the distribution of this parameter. Bin with is 0.2 Å.

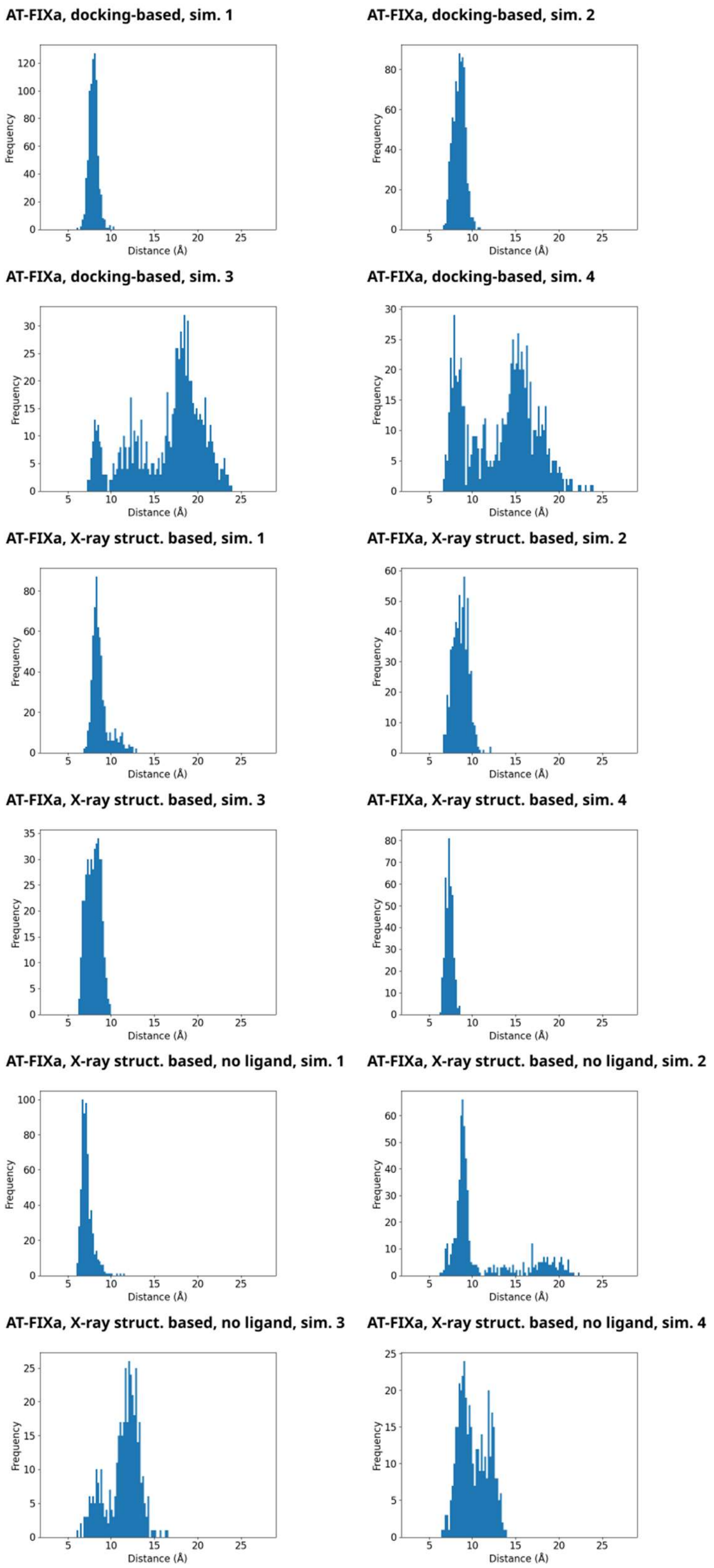

Supplementary Figure S6. Histograms for the Arg150 (FIXa) – Tyr253 (AT) distances in the AT–FIXa complex simulations, showing the distribution of this parameter. Bin with is 0.2 Å.

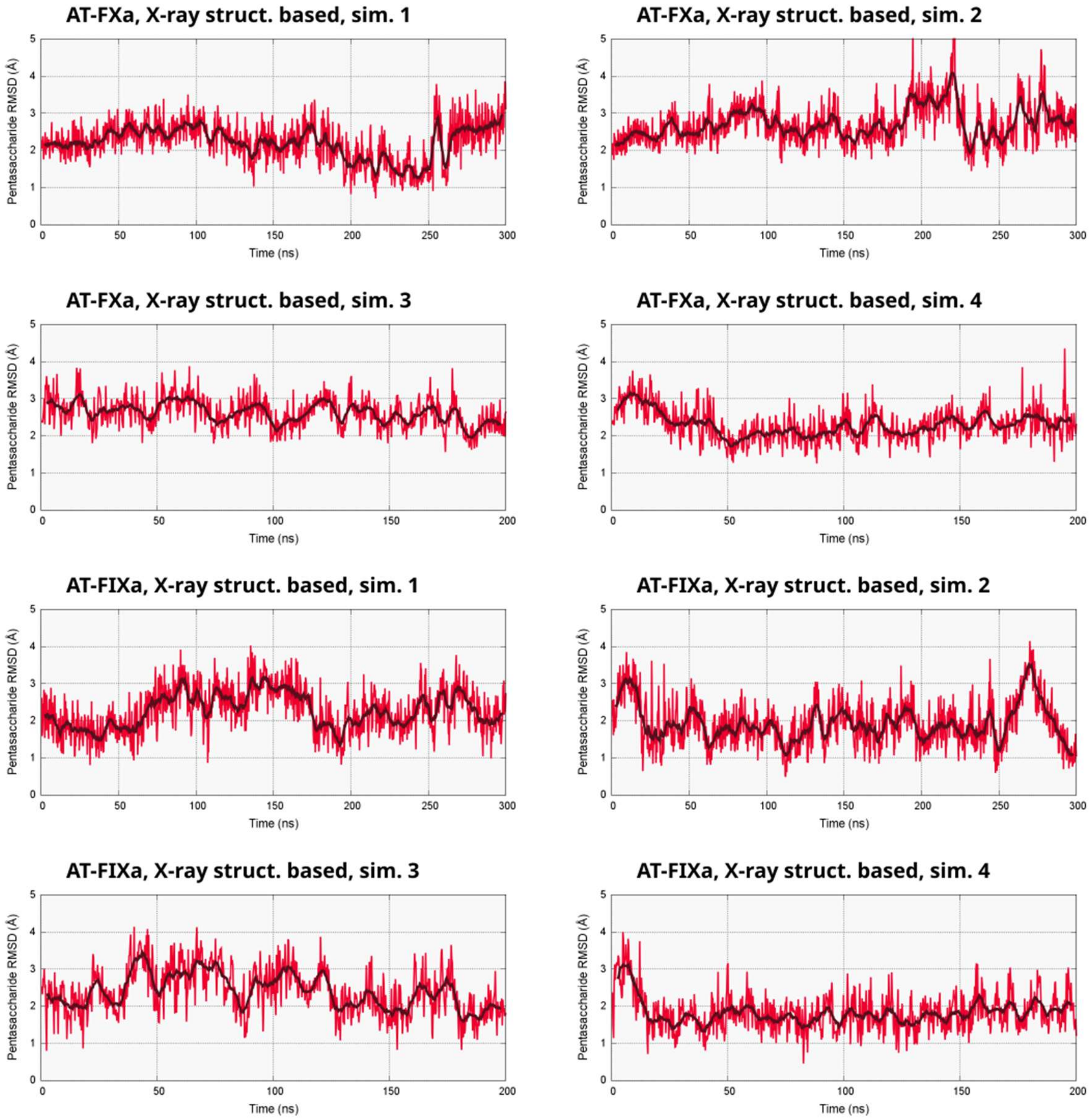

Supplementary Figure S7. The RMSD of the pentasaccharide ligand compared to its position in the energy minimized structure, as a function of time, in the X-ray diffraction based simulations with a pentasaccharide.

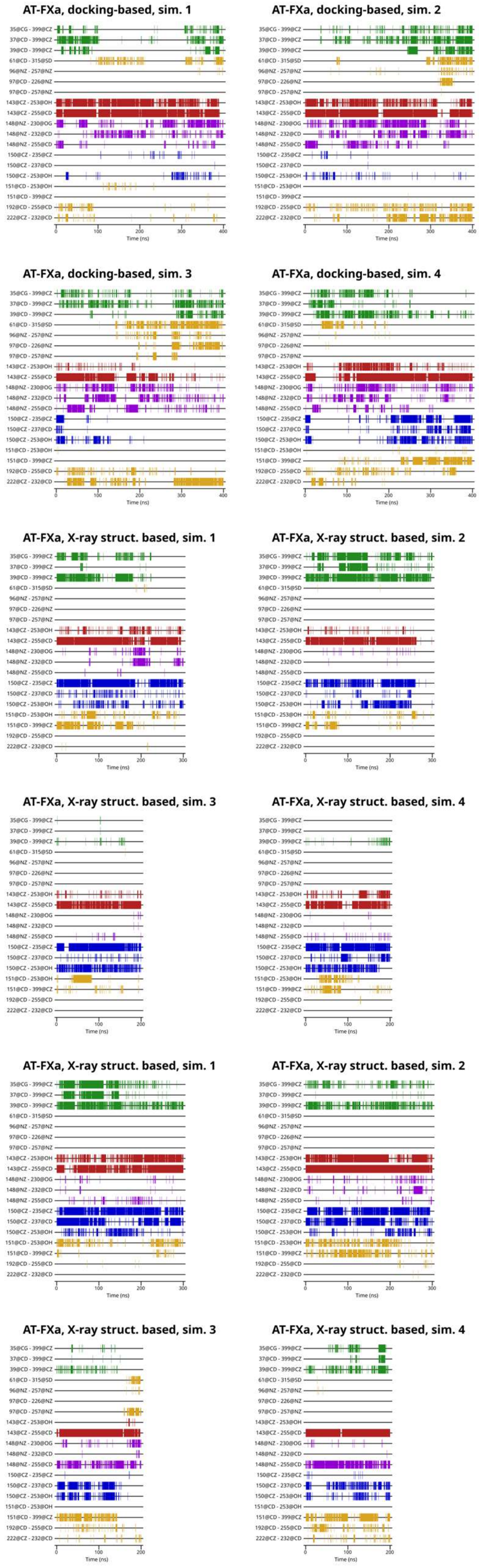

Supplementary Figure S8 The amino acids involved in exosite interactions, as a function of time, in all simulations of the AT-FXa complex. The first number is the residue in FXa, while the second is the amino acid in AT. A residue pair is shown as "interacting" if the distance is below a cut-off value of 6 Å

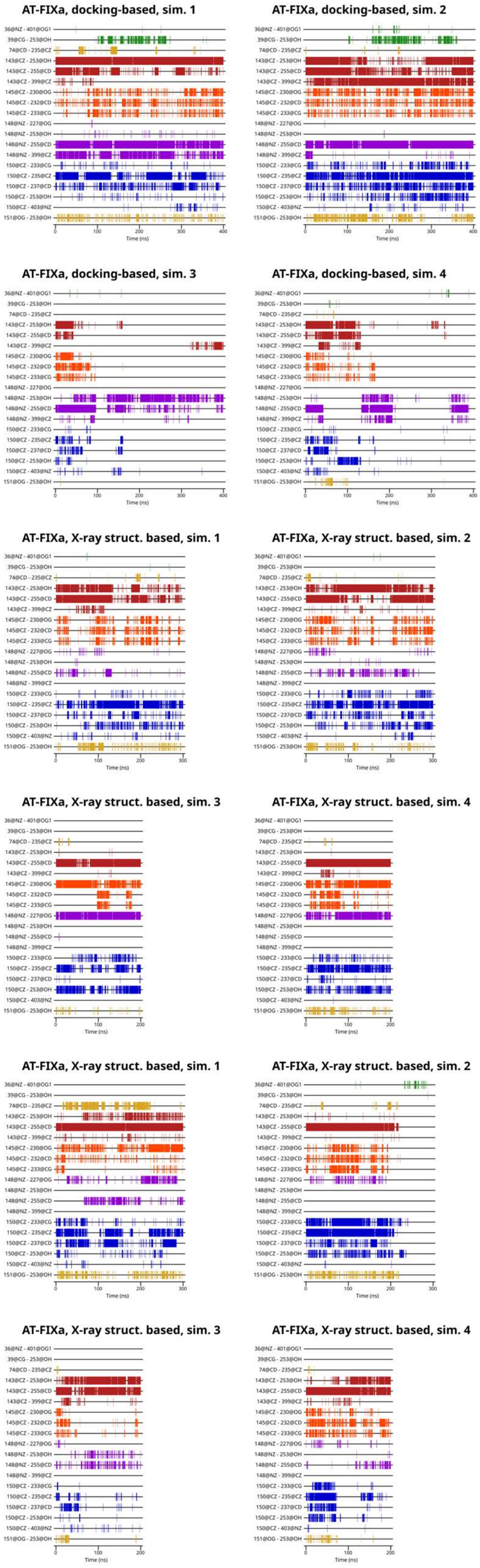

Supplementary Figure S9. The amino acids involved in exosite interactions, as a function of time, in all simulations of the AT-FIXa complex. The first number is the residue in FIXa, while the second is the amino acid in AT. A residue pair is shown as “interacting” if the distance is below a cut-off value of 6 Å

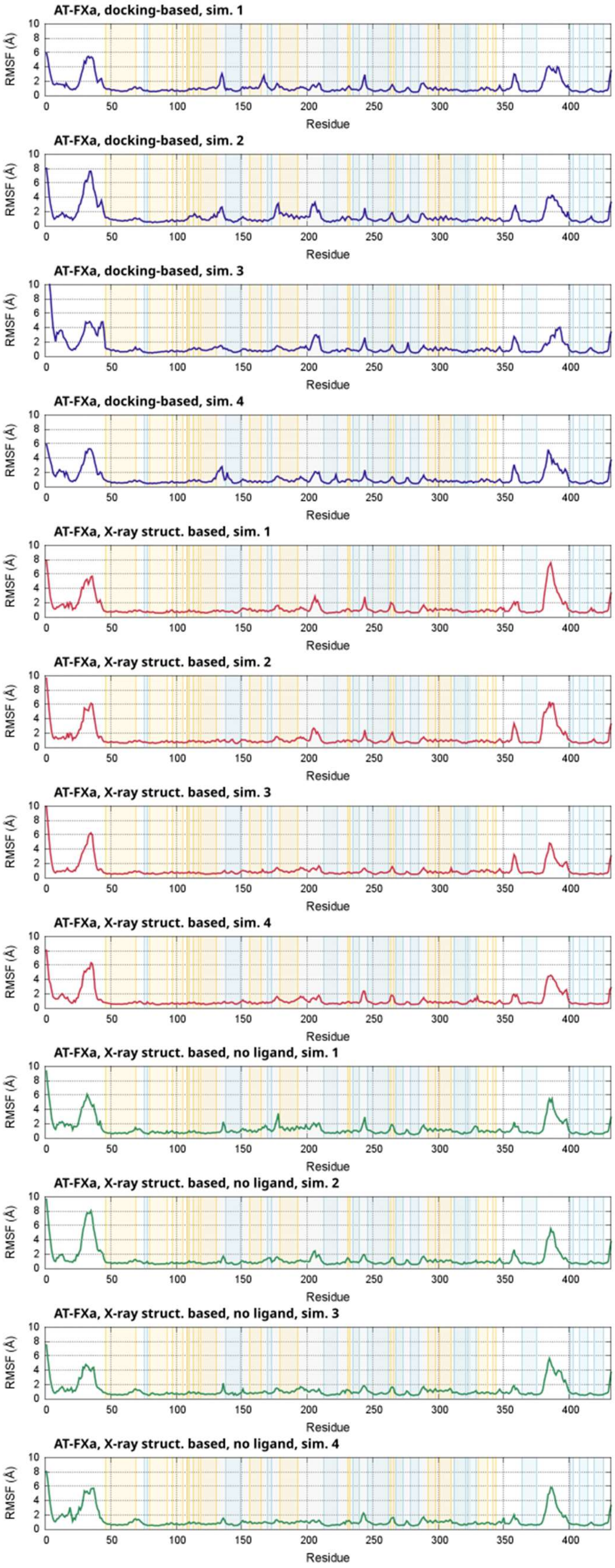

Supplementary Figure S10 . . RMSF analysis for the α-carbon atoms of AT, in the simulations of AT-FXa complex.

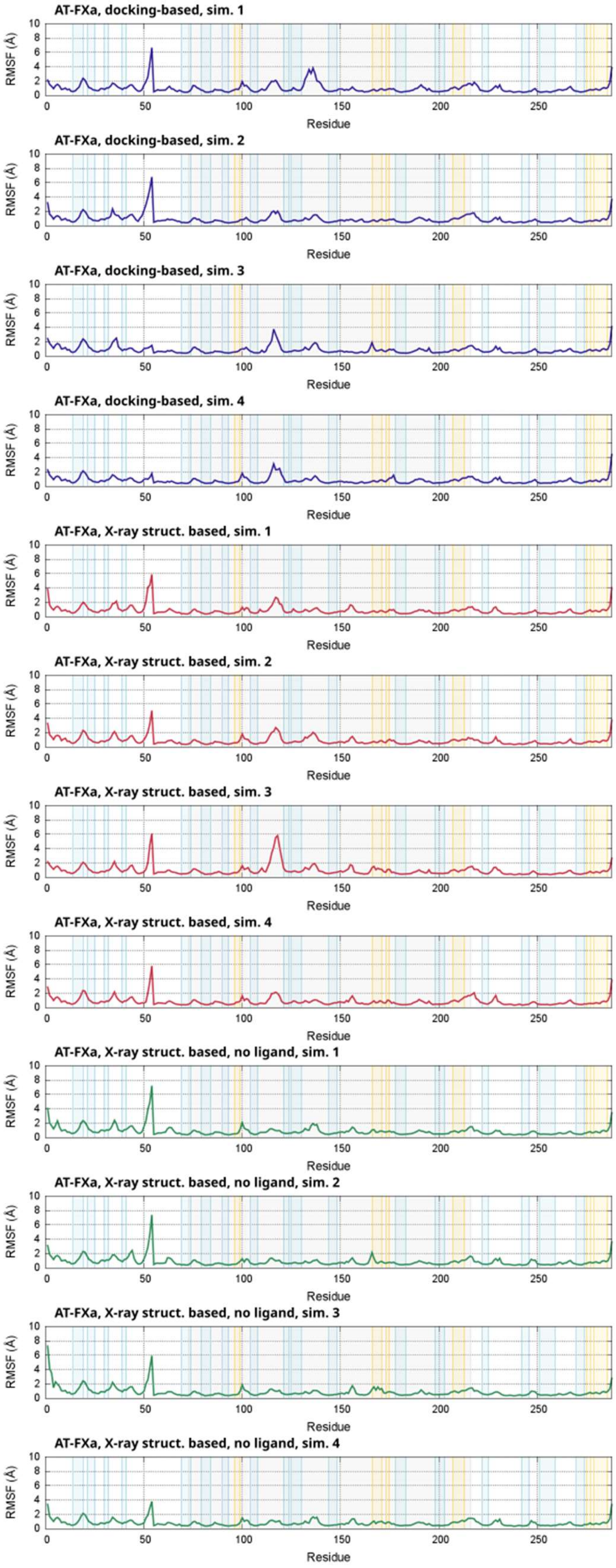

Supplementary Figure S11. RMSF analysis for the α-carbon atoms of FXa, in the simulations of AT-FXa complex.

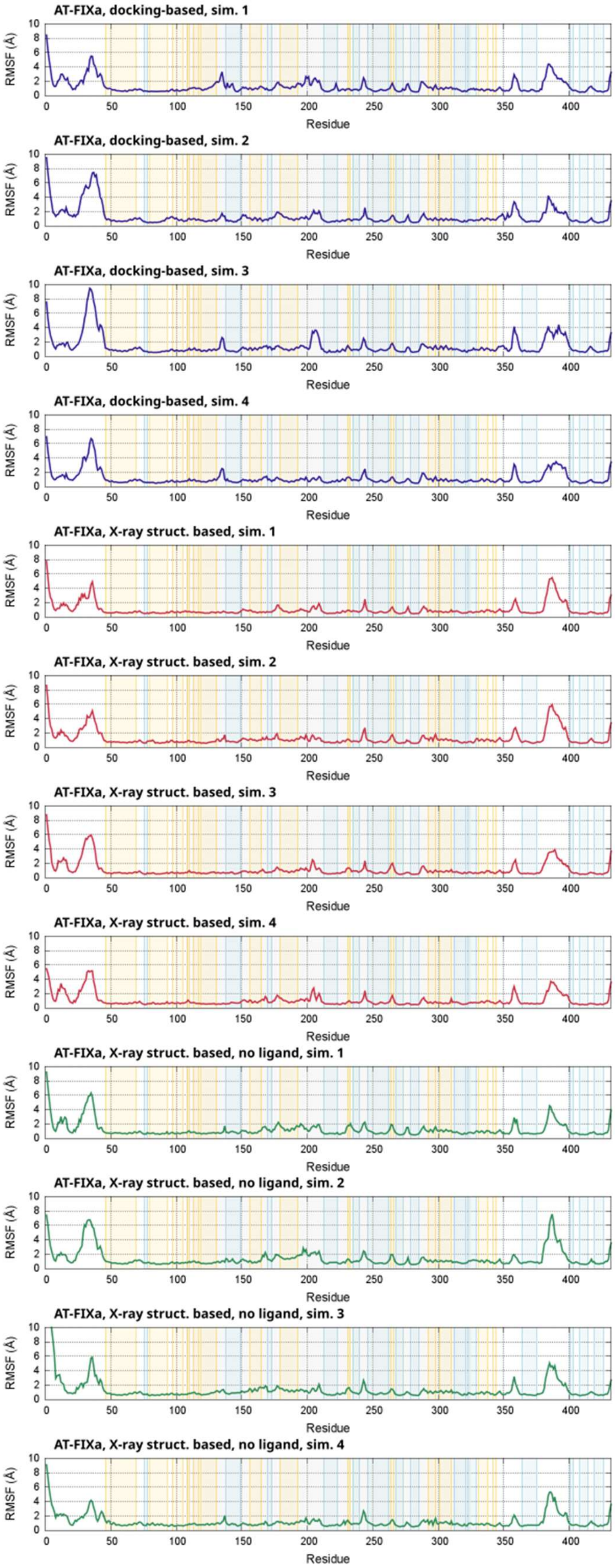

Supplementary Figure S12. RMSF analysis for the α-carbon atoms of AT, in the simulations of AT-FIXa complex.

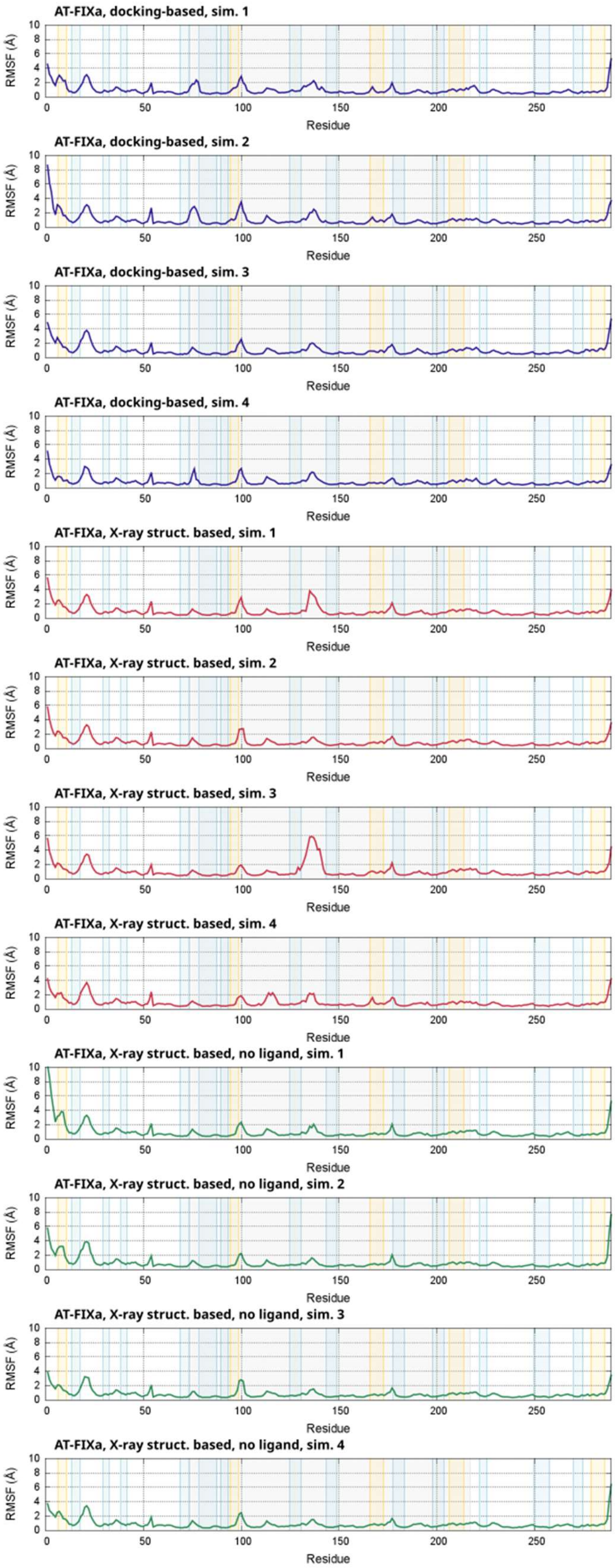

Supplementary Figure S13. RMSF analysis for the α-carbon atoms of FIXa, in the simulations of AT-FIXa complex.

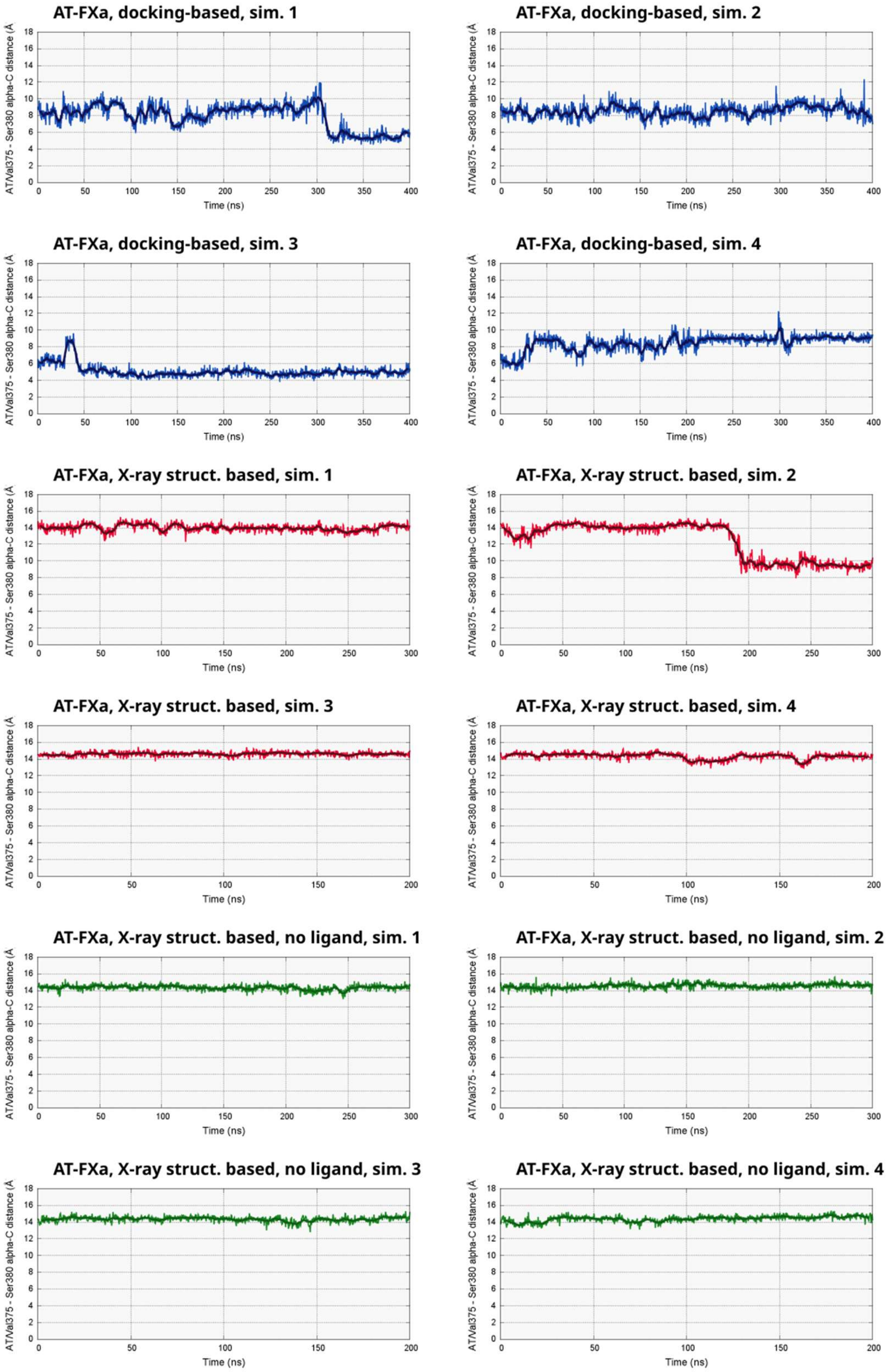

Supplementary Figure S14. The distance between the α-carbon atoms of AT residues Val375 and Ser380 in the simulations of the AT-FXa complex, as function of time. Lower values correspond to "inserted" conformations of the hinge region.

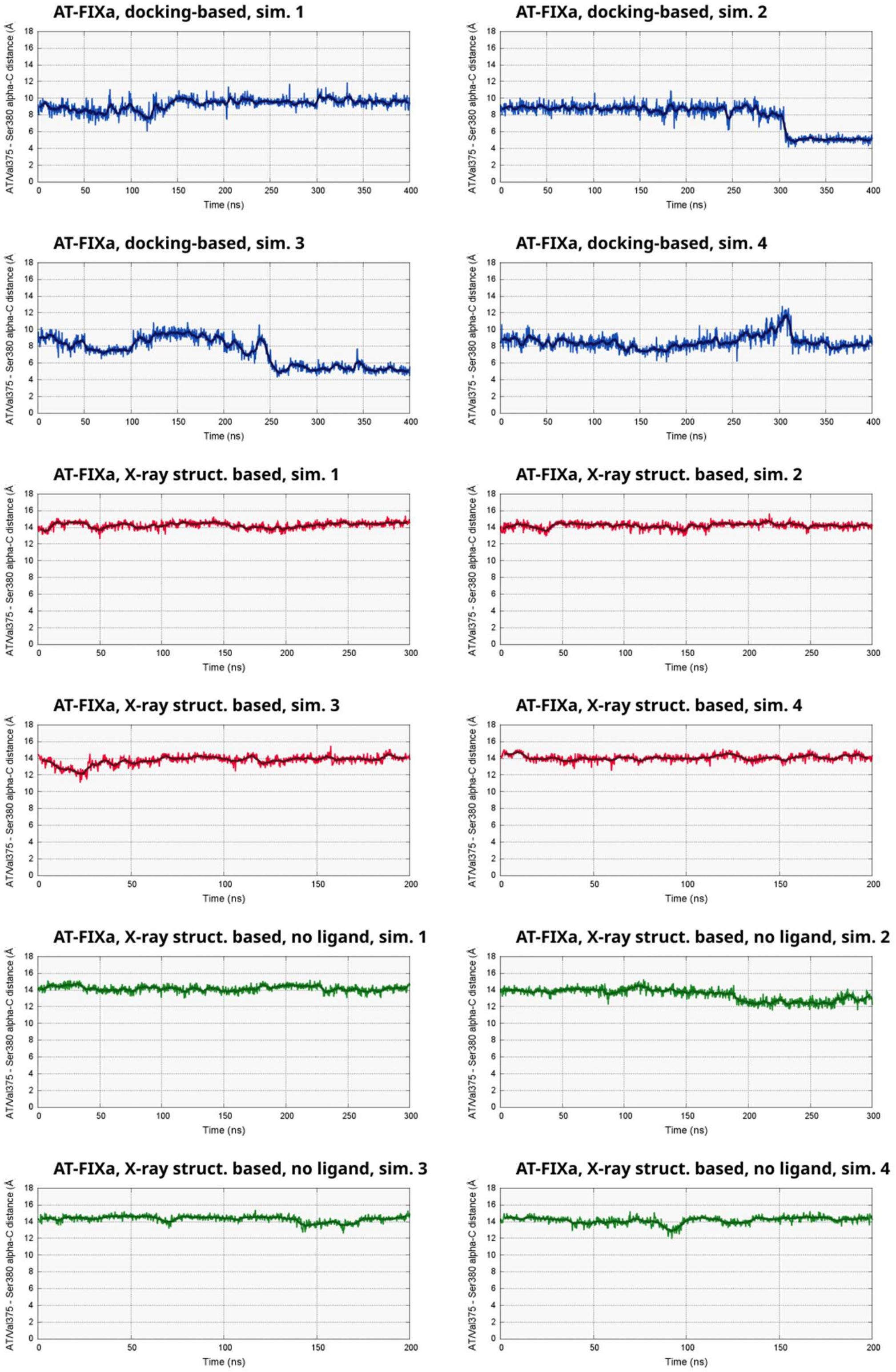

Supplementary Figure S15. The distance between the α-carbon atoms of AT residues Val375 and Ser380 in the simulations of the AT-FIXa complex, as function of time. Lower values correspond to "inserted" conformations of the hinge region.

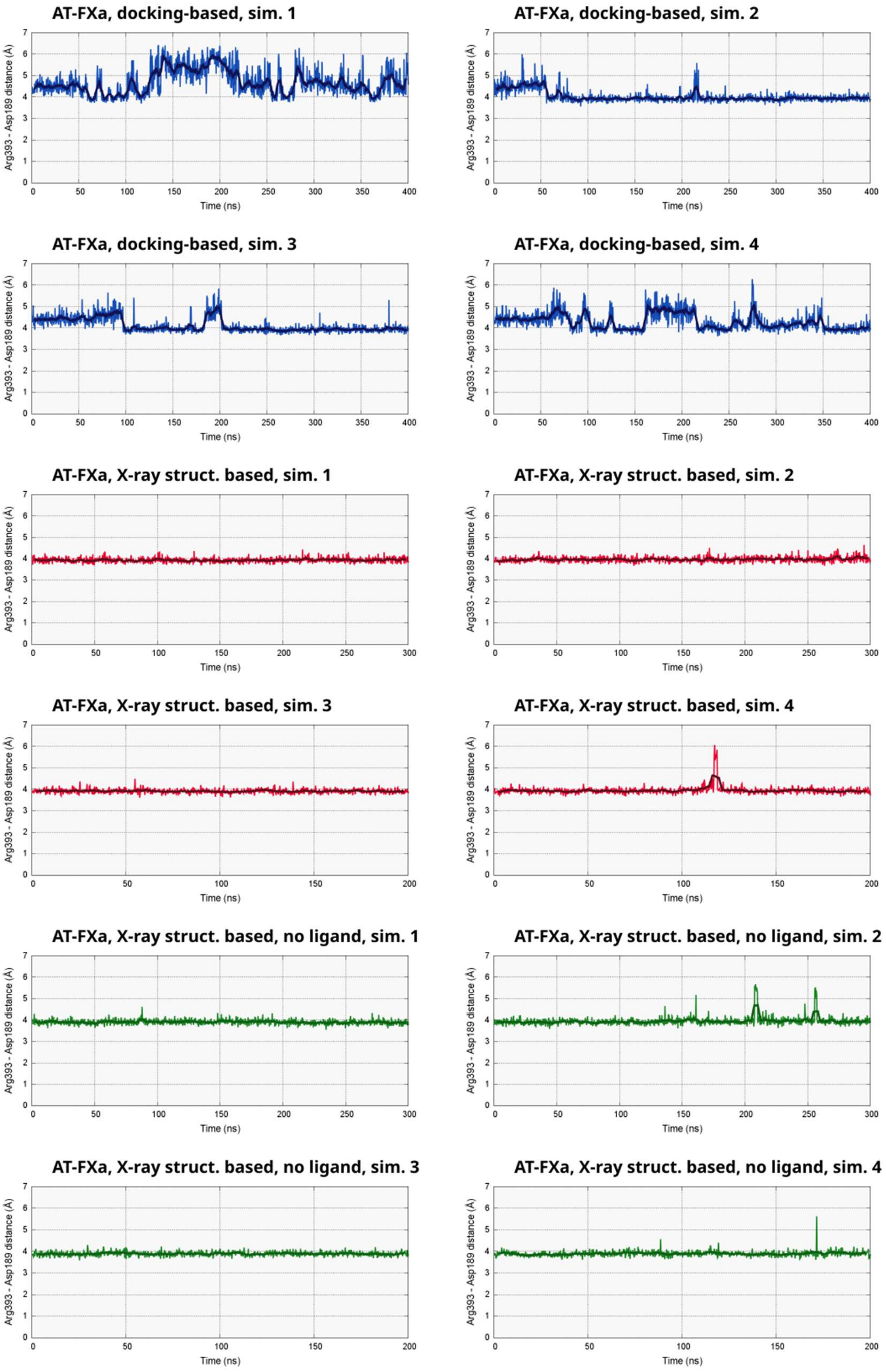

Supplementary Figure S16. Distance between the side chains of Arg393 (AT) and Asp189 (FXa), as a function of time, in the simulations of the AT-FXa complex.

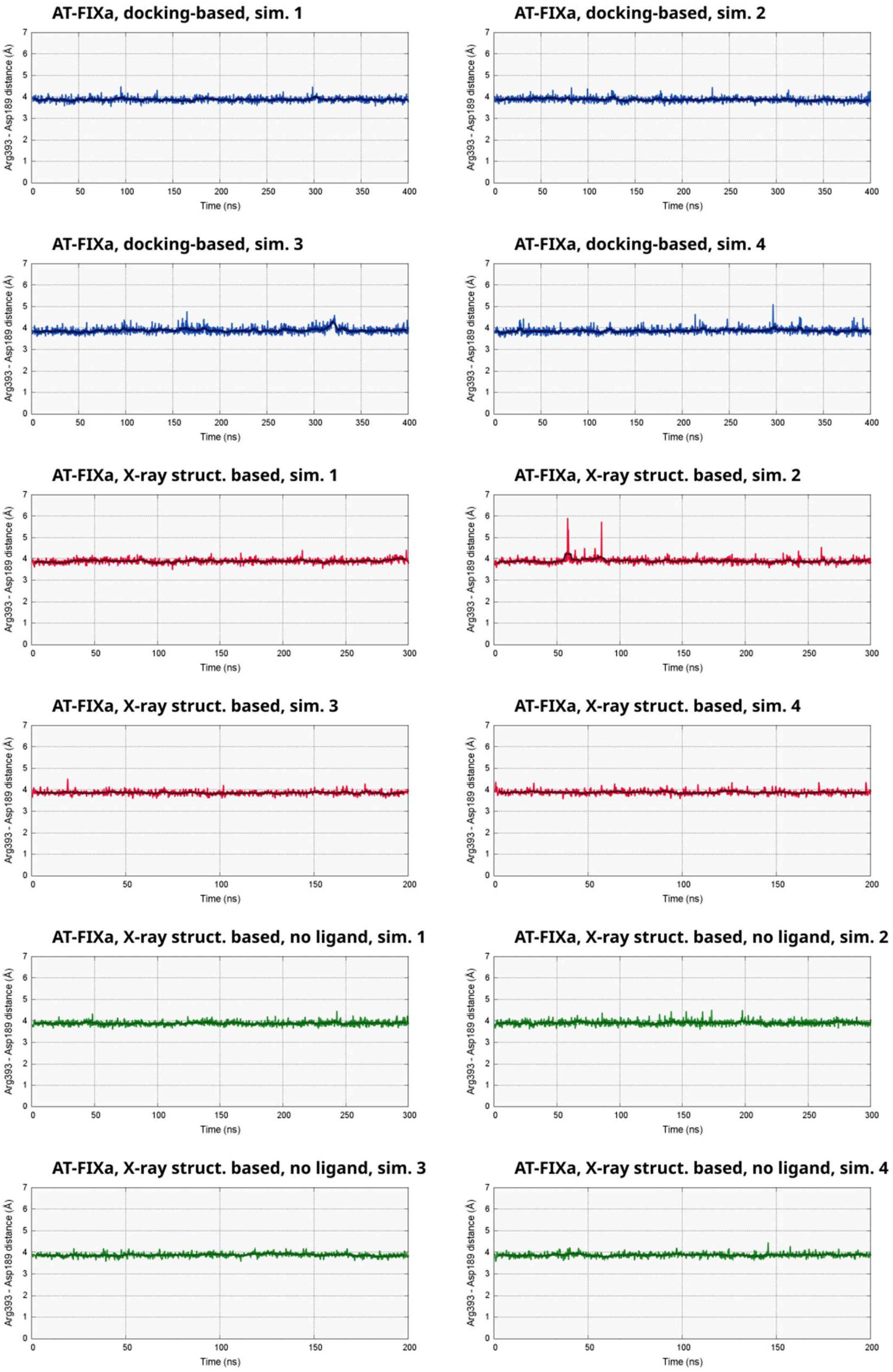

Supplementary Figure S17. Distance between the side chains of Arg393 (AT) and Asp189 (FIXa), as a function of time, in the simulations of the AT-FIXa complex.

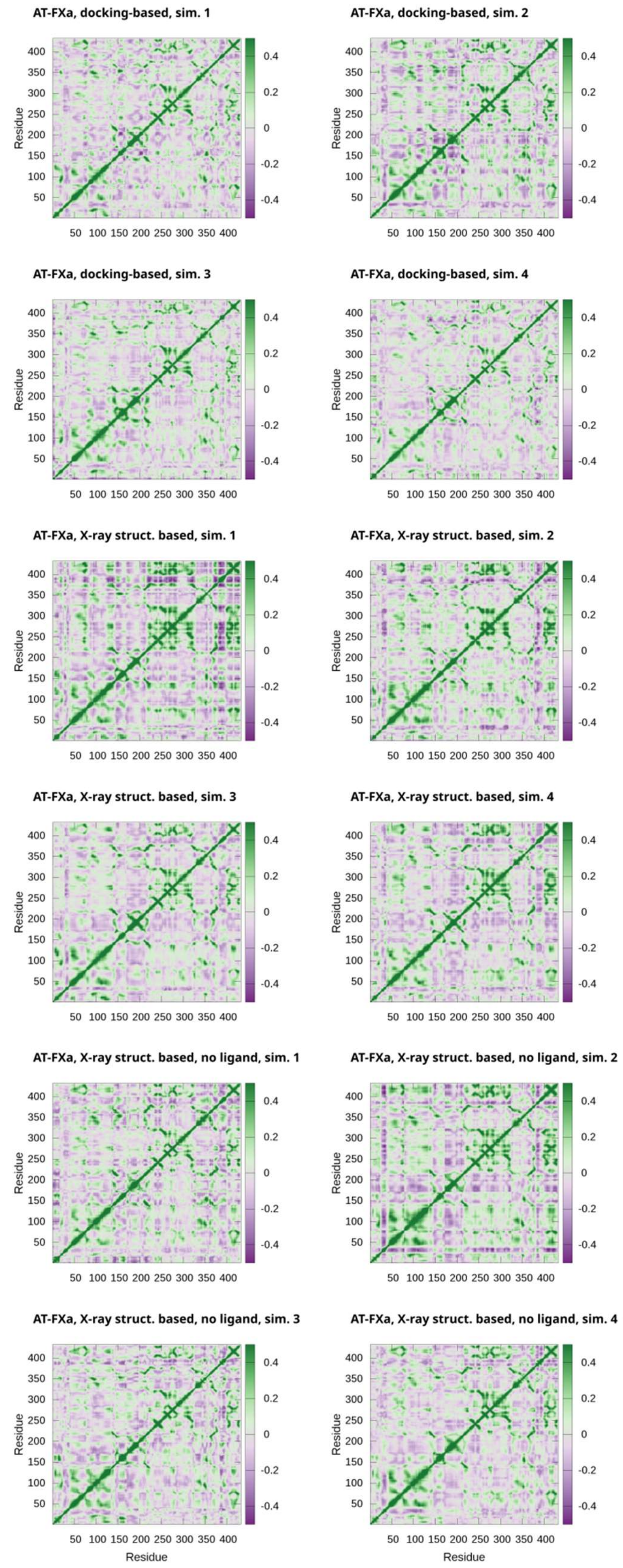

Supplementary Figure S18. Dynamic cross-correlation matrices (DCCM) for the simulations of the AT-FXa complex.

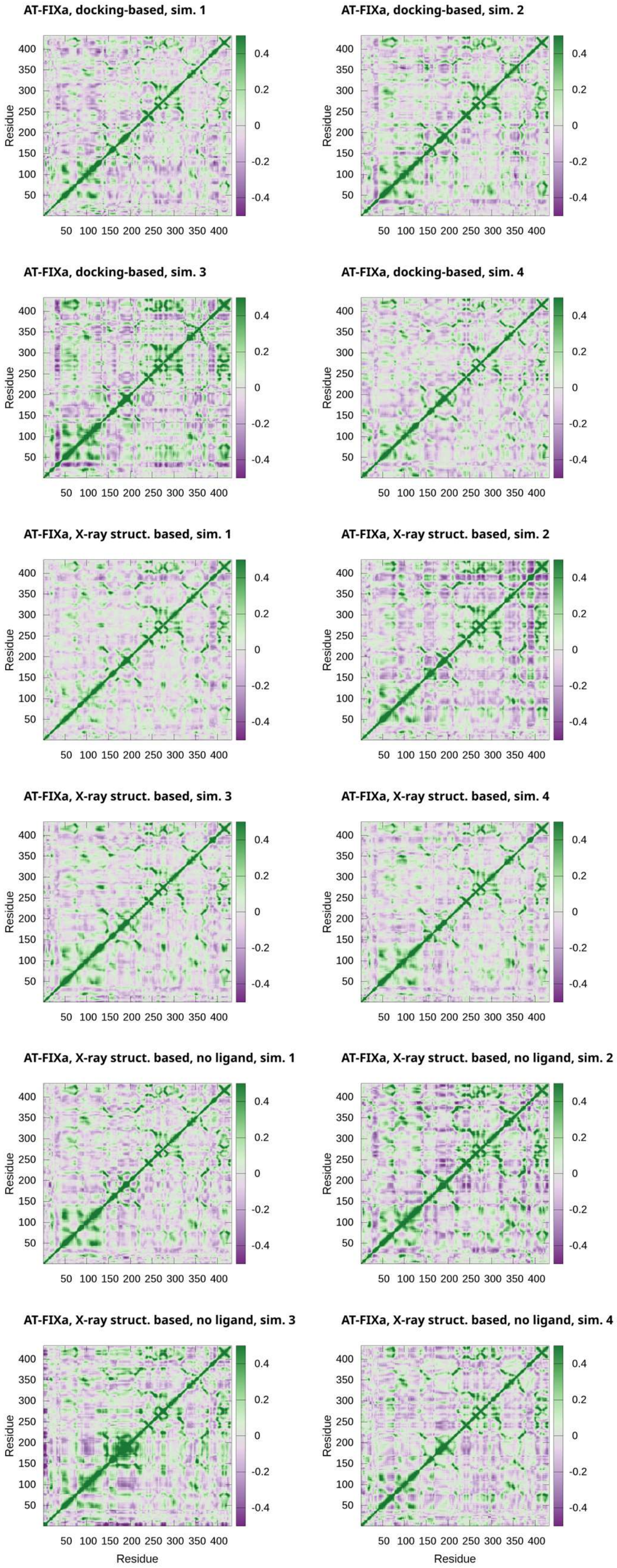

Supplementary Figure S19 Dynamic cross-correlation matrices (DCCM) for the simulations of the AT-FIXa complex.

**AT-FXa, based on docking**	150 ns equilibrium MD, exosite interaction restrained	100 ns equilibrium MD, no restraint	100 ns GaMD equilibration	2 × 400 ns production GaMD
			100 ns GaMD equilibration	2 × 400 ns production GaMD
**AT-FXa, based on X-ray structure**	150 ns equilibrium MD simulation		100 ns GaMD equilibration	2 × 300 ns production GaMD
			100 ns GaMD equilibration	2 × 200 ns production GaMD
**AT-FXa, based on X-ray structure, no ligand**	150 ns equilibrium MD simulation		100 ns GaMD equilibration	2 × 300 ns production GaMD
			100 ns GaMD equilibration	2 × 200 ns production GaMD
**AT-FIXa, based on docking**	150 ns equilibrium MD, exosite interaction restrained	100 ns equilibrium MD, no restraint	100 ns GaMD equilibration	2 × 400 ns production GaMD
			100 ns GaMD equilibration	2 × 400 ns production GaMD
**AT-FIXa, based on X-ray structure**	150 ns equilibrium MD simulation		100 ns GaMD equilibration	2 × 300 ns production GaMD
			100 ns GaMD equilibration	2 × 200 ns production GaMD
**AT-FIXa, based on X-ray structure, no ligand**	150 ns equilibrium MD simulation		100 ns GaMD equilibration	2 × 300 ns production GaMD
			100 ns GaMD equilibration	2 × 200 ns production GaMD

Supplementary Table S1. Summary of the simulations performed. The earliest steps of MD protocol – minimization, temperature and pressure equilibration – are not shown.

.

AT-FXa, docking-based	AT-FXa, X-ray diffraction based	AT-FXa, X-ray diffraction based, no ligand
41 - 394		
41 - 395	35 - 397	35 - 397
42 - 394	41 - 394	41 - 395
61 - 396	41 - 395	41 - 397
147 - 391	41 - 397	42 - 394
191 - 393	42 - 394	58 - 394
192 - 392	58 - 394	61 - 396
192 - 393	191 - 393	61A - 397
192 - 394	192 - 393	97 - 384
192 - 395	192 - 394	97 - 385
193 - 393	192 - 395	98 - 385
193 - 394	193 - 393	191 - 393
193 - 395	193 - 394	192 - 393
194 - 393	193 - 395	192 - 394
195 - 393	194 - 393	192 - 395
195 - 394	195 - 393	193 - 393
215 - 391	195 - 394	193 - 394
216 - 390	214 - 392	193 - 395
216 - 391	214 - 393	195 - 393
216 - 392	215 - 391	195 - 394
217 - 388	215 - 392	214 - 393
217 - 389	215 - 393	215 - 391
217 - 390	216 - 390	215 - 392
217 - 392	216 - 391	215 - 393
219 - 389	217 - 389	216 - 391
219 - 390	217 - 390	217 - 391
219 - 391	219 - 388	219 - 390
219 - 392	219 - 389	219 - 391
220 - 391		

AT-FIXa, docking-based	AT-FIXa, X-ray diffraction based	AT-FIXa, X-ray diffraction based, no ligand
	35 - 397	35 - 397
	36 - 397	36 - 397
	36 - 398	36 - 398
	38 - 397	38 - 397
38 - 397	41 - 394	41 - 394
41 - 397	41 - 395	41 - 395
58 - 395	41 - 396	41 - 397
58 - 396	41 - 397	42 - 394
191 - 393	42 - 394	191 - 393
192 - 393	58 - 394	192 - 393
192 - 394	173 - 388	192 - 394
192 - 395	174 - 388	192 - 395
214 - 394	191 - 393	193 - 393
215 - 393	192 - 393	193 - 394
215 - 394	192 - 394	193 - 395
216 - 392	192 - 395	195 - 393
216 - 393	193 - 393	195 - 394
217 - 392	193 - 394	214 - 392
219 - 391	193 - 395	214 - 393
219 - 392	195 - 393	215 - 391
219 - 393	195 - 394	215 - 392
	214 - 393	215 - 393
	215 - 391	216 - 390
	215 - 392	216 - 391
	215 - 393	217 - 390

216 - 391
217 - 391
219 - 390
219 - 391
221A - 383

Supplementary Table S2. Interaction of the RCL of AT with the two factors. The distances were cal-culated for the α-carbon atoms in the representative frames of the cluster analysis. A distance cutoff of 6 Å was used.
